# Dysregulation of gut microbiota stimulates NETs-driven HCC intrahepatic metastasis: therapeutic implications of healthy faecal microbiota transplantation

**DOI:** 10.1080/19490976.2025.2476561

**Published:** 2025-03-18

**Authors:** Zhe Deng, Si Mei, Zhaoguang Ouyang, Ruoyu Wang, Lihuai Wang, Bo Zou, Jingjing Dai, Kexin Mao, Qian Li, Qianqian Guo, Chun Yi, Fanying Meng, Mingxia Xie, Xue Zhang, Rongrong Wang, Tianhao Deng, Zhenyu Wang, Xiaozheng Li, Qing Wang, Bin Liu, Xuefei Tian

**Affiliations:** a College of Integrated Chinese and Western Medicine, Hunan University of Chinese Medicine, Changsha, Hunan, China; b Sidney Kimmel Comprehensive Cancer Center at Johns Hopkins, Baltimore, MD, USA; c Hunan Province University Key Laboratory of Oncology of Traditional Chinese Medicine, Changsha, Hunan, China; d Key Laboratory of Traditional Chinese Medicine for Mechanism of Tumor Prevention &Treatment, Changsha, Hunan, China; e Department of Physiology, Faculty of Medicine, Hunan University of Chinese Medicine, Changsha, Hunan, China; f School and Hospital of Stomatology, Tianjin Medical University, Tianjin, China; g The First Hospital of Hunan University of Chinese Medicine, Changsha, Hunan, China; h College of Traditional Chinese Medicine, Hunan University of Chinese Medicine, Changsha, Hunan, China; i Department of Pathology, Faculty of Medicine, Hunan University of Chinese Medicine, Changsha, Hunan, China; j College of Pharmacy, Hunan University of Chinese Medicine, Changsha, Hunan, China; k Hunan Province Integrated Traditional Chinese and Western Medicine Hospital, Changsha, Hunan, China; l JCY Biotech Ltd., Pingshan Translational Medicine Center, Shenzhen Bay Laboratory, Shenzhen, China; m College of Life Sciences and Oceanography, Shenzhen University, Shenzhen, China; n College of Biology, School of Biomedical Sciences, Hunan University, Changsha, China; o Shanghai OE Biotech Co. Ltd, Shanghai, China

**Keywords:** Hepatocellular carcinoma, intrahepatic metastasis, human flora-associated mouse model, neutrophil extracellular traps, healthy faecal microbiota transplantation

## Abstract

The stringent regulation of intrahepatic metastases is essential for improving survival outcomes in patients with hepatocellular carcinoma (HCC). This study investigated the impact of gut microbiota on intrahepatic metastasis of HCC and evaluated the therapeutic potential of healthy fecal microbiota transplantation (FMT). Dysregulation of the gut microbiota, characterized by a significant reduction in the abundance of beneficial bacteria, such as *Anaerotruncus colihominis* and *Dysosmobacter welbionis*, was observed in patients with intrahepatic metastatic HCC. A human flora-associated (HFA) intrahepatic metastatic HCC mouse model was successfully established through consecutive 4 weeks of human-mouse FMT. Dysregulation of gut microbiota promoted intrahepatic metastasis in the mouse model, primarily by enhancing neutrophil-mediated inflammatory responses and lead to excessive formation of neutrophil extracellular traps (NETs). Consequently, it promoted tumor vascular growth and tissue necrosis, resulting in intrahepatic metastasis of HCC. Notably, FMT from healthy donors mitigated these pathological processes. This study elucidated the role and mechanism of dysregulated gut microbiota in promoting intrahepatic metastasis of HCC. Healthy FMT emerges as a promising novel therapeutic strategy for preventing and treating intrahepatic metastasis of HCC.

## GRAPHICAL ABSTRACT

**Figure:**
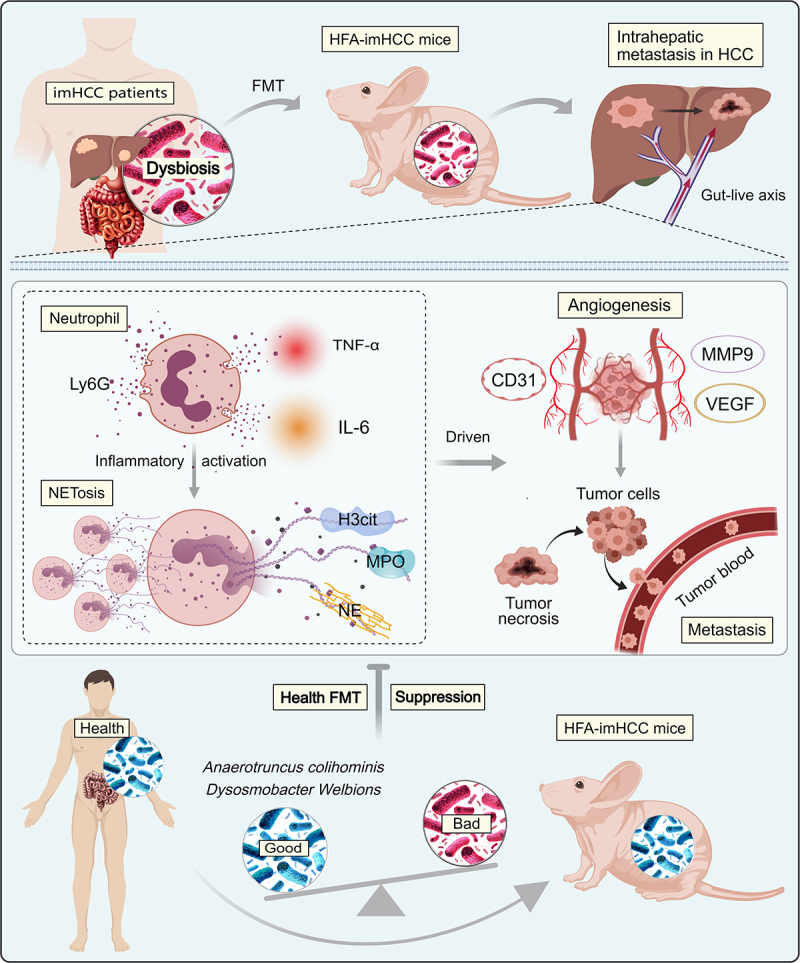

## 1. Introduction

Hepatocellular carcinoma (HCC) constitutes approximately 90% of primary liver cancers.^1–3^ Nearly 80% of HCC cases and approximately 30% of surgically resected small HCC (<3 cm) cases exhibit intrahepatic metastasis at an earlier stage and higher rate than extrahepatic metastasis.^4–6^ The gut microbiota, which is connected to the liver through the portal vein, forms the gut-liver axis. Dysbiosis of the gut microbiota has been established as a crucial determinant of the occurrence and development of HCC.^7–9^ However, the exact role of dysregulated gut microbiota in intrahepatic metastasis of HCC remains unclear.

Recently, several studies have indicated that tumor-associated neutrophil extracellular traps (NETs) can stimulate tumor growth, promote angiogenesis, facilitate invasion and metastasis, and induce tumor-related thrombosis.^10–12^ The liver recruits neutrophils from the bloodstream in response to bacteria and their metabolites. These neutrophils then generate NETs to capture and eliminate bacteria.^13,14^ NETs are chromatin filament structures wrapped in citrullinated histone H3 (CitH3), neutrophil elastase (NE), myeloperoxidase (MPO), and cytoplasmic proteins. Elevated neutrophil-to-lymphocyte ratio (NLR) and MPO-DNA markers have been identified as markers of advanced HCC, poor prognosis, and inadequate treatment response.^15,16^ Although studies have confirmed that gut microbiota can activate excessive formation of NETs,^17,18^ the potential interrelation among gut microbiota, excessive NETs activation, and intrahepatic metastasis of HCC remains unknown.

In this study, we opted to demonstrate the dysregulation of gut microbiota in patients with intrahepatic metastatic HCC. By establishing a human flora-associated (HFA) intrahepatic metastatic HCC mouse model, we discovered the crucial role of gut microbiota disequilibrium in promoting intrahepatic metastasis of HCC by stimulating excessive NETs formation. Furthermore, we demonstrated that healthy fecal microbiota transplantation (FMT) effectively suppressed the intrahepatic metastasis of HCC. Overall, our findings highlight the pivotal role of dysregulated gut microbiota in intrahepatic metastasis of HCC and indicate a therapeutic strategy based on microbial interventions for HCC.

## 2. Results

### 2.1. Dysregulation of the gut microbiota in patients with intrahepatic metastatic HCC

**Figure 1.**
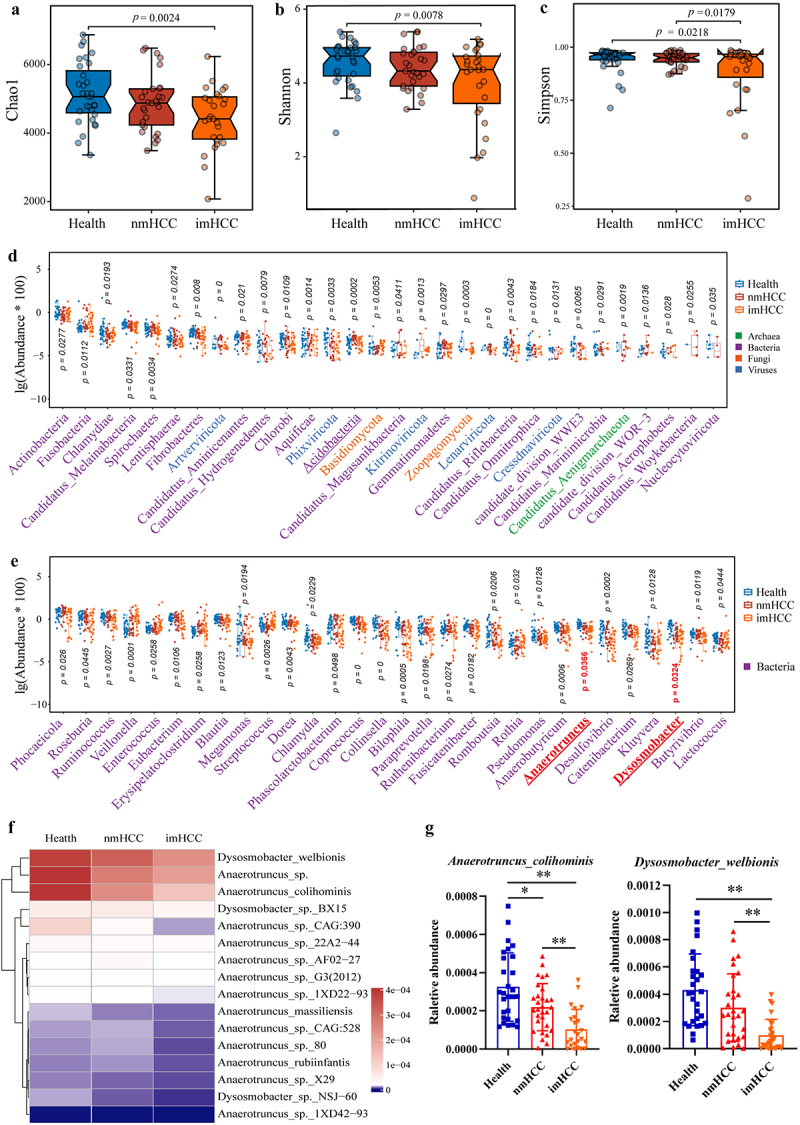
Dysregulation of the gut microbiota in patients with intrahepatic metastatic HCC. (a-c). Alpha diversity analysis of the gut microbiota in three cohorts: healthy individuals, patients with nmHCC, and patients with imHCC (a). Chao1 index; (b). Shannon index; (c). Simpson index); (d-e). Differential microbiota at the phylum (d) and genus (e) levels among the top 30 taxa in the three cohorts. (f). Relative abundance heatmap of all species of *Anaerotruncus* and *Dysosmobacter* in the gut microbiota of the three cohorts. (g). Differential analysis of the relative abundance of *Anaerotruncus colihominis* and *Dysosmobacter welbionis* among the three cohorts. HCC: Hepatocellular carcinoma, health: healthy individuals, nmHCC: patients with non-metastatic HCC, imHCC: patients with intrahepatic metastatic HCC.

To evaluate alteration in the gut microbiota of patients with intrahepatic metastatic HCC, we conducted a comparative analysis of three cohorts: patients with intrahepatic metastatic HCC (imHCC) (*n* = 28), patients with non-metastatic HCC (nmHCC) (*n* = 26), and healthy control individuals (*n* = 30) (Table S1). Intrahepatic metastatic HCC, multi-focal HCC, and non-metastatic HCC was differentiated according to the imaging characteristics from unenhanced and contrast-enhanced magnetic resonance imaging (MRI) of the upper abdomen (Figure S1). The NLR gradually increased according to the sequence of the healthy, nmHCC, and imHCC groups, suggesting a potential correlation between intrahepatic metastasis of HCC and alterations in the NLR (Table S1). The metagenomic sequencing results revealed a decreasing trend in alpha diversity (Chao1, Shannon, and Simpson indices) in the gut microbiota of the nmHCC and imHCC groups, compared to the healthy group. The most significant decrease in alpha diversity (*p* < 0.05) was observed in the imHCC group (Figure 1(a-c)). Among the top 30 differentially abundant microbial taxa at the phylum and genus levels, the imHCC group showed a substantial decrease in the abundance of 86.67% (26/30) and 90% (27/30) of these taxa compared to the healthy and nmHCC groups. The abundances of various core beneficial genera in the gut microbiota, such as *Phocaeicola* within *Bacteroides*, *Roseburia*, *Enterococcus*, *Eubacterium*, *Dorea*, *Coprococcus*, *Anaerotruncus*, *Dysosmobacter*, and *Butyrivibrio* within *Firmicutes*, were significantly decreased in the imHCC group (Figure 1(d-e)). Compared to the healthy and nmHCC groups, the abundance of all bacterial species within the genera, *Anaerotruncus* and *Dysosmobacter*, displayed a declining trend in the imHCC group (Figure 1(f)). Relative quantitative analysis revealed a significant decrease in the abundance of *Anaerotruncus colihominis* and *Dysosmobacter welbionis* in the imHCC group, which constituted a relatively high proportion in the *Anaerotruncus* and *Dysosmobacter* (Figure 1(g)). These findings suggest a substantial reduction in the diversity and richness of the gut microbiota in patients with intrahepatic metastatic HCC. The reduced abundances of *Anaerotruncus colihominis* and *Dysosmobacter welbionis* may play a crucial role in triggering intrahepatic metastasis of HCC.

### 2.2. Construction of the HFA intrahepatic metastatic HCC mouse model

**Figure 2.**
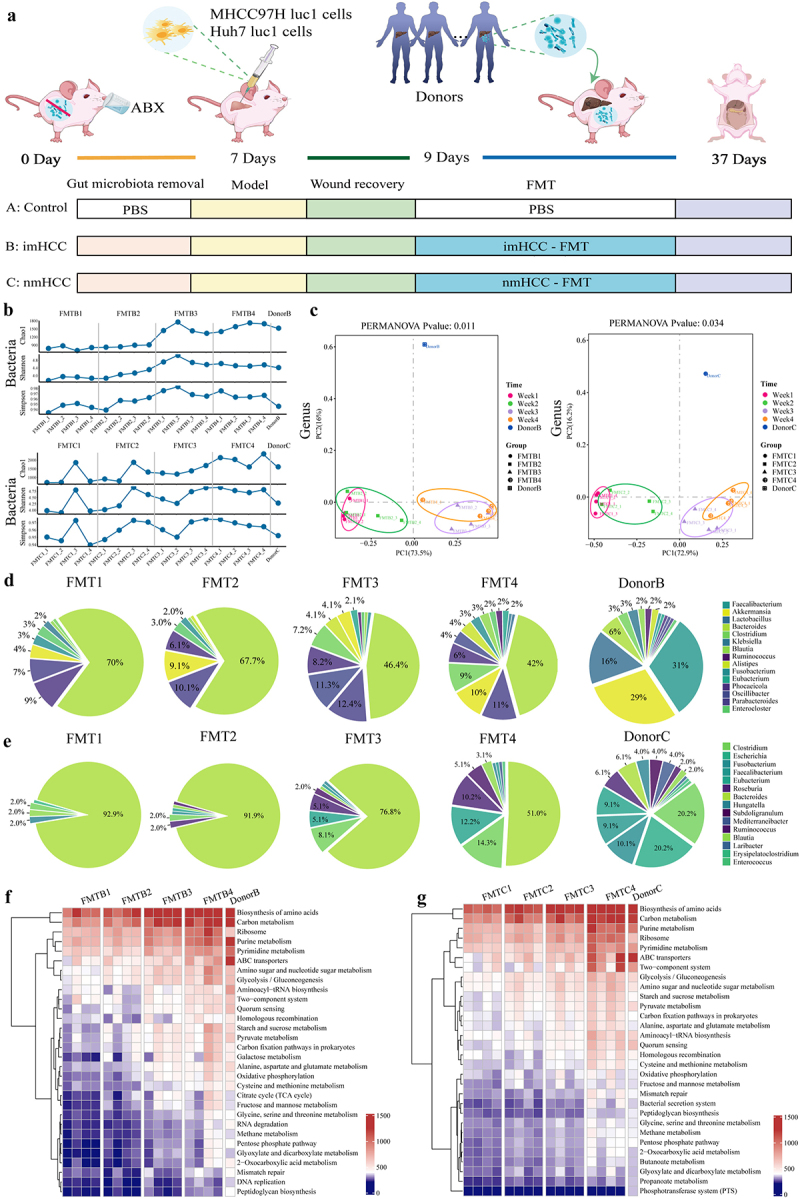
Construction of a human flora-associated (HFA) intrahepatic metastatic HCC mouse model. (a). Experimental design workflow. (b). Dynamic changes in the alpha diversity of the gut microbiota in recipient mice in the imHCC and nmHCC groups after FMT. (c). Dynamic changes in the principal coordinates analysis (PCoA) of the gut microbiota in recipient mice in the imHCC and nmHCC groups after FMT. (d-e). Dynamic changes in the relative abundance of the top 15 genera from imHCC (d) and nmHCC donors (e) in the gut of corresponding recipient mice after FMT. F-G. Dynamic changes in the top 30 gene functions of the gut microbiota from imHCC (f) and nmHCC donors (g) in the gut of corresponding recipient mice after FMT. HCC: hepatocellular carcinoma; imHCC: intrahepatic metastatic HCC; nmHCC: non-metastatic HCC. Donor B: donors with imHCC (*n* = 28); donor C: donors with nmHCC (*n* = 26). FMT: fecal microbiota transplantation; FMT1–4 represents the transplantation time for each week.

To elucidate the effect of dysbiotic gut microbiota on intrahepatic metastasis of HCC at the animal level, we established an HFA intrahepatic metastatic HCC mouse model. Briefly, an antibiotic cocktail (ABX) was administered to deplete the intestinal commensal bacteria of mice (Figure S2). The human liver cancer cell lines MHCC97H Luc1 and Huh-7 Luc1 were injected into the spleen of mice to enable cancer cells to metastasize to the liver via the portal vein. Two days post-injection, the surgical wounds of the mice had stabilized, the gut microbiota from patients with intrahepatic metastatic HCC and non-metastatic HCC were transplanted into mice. Faecal samples from recipient mice were collected weekly to monitor the colonization of human-sourced microbiota (Figure 2(a)). The alpha diversity of the gut microbiota in both groups of FMT recipient mice showed significant fluctuations in week 3 post-FMT, gradually approaching the donor profile in week 4 while maintaining stability (Figure 2(b)). PCoA (beta diversity) revealed a distinct separation in the gut microbiota community structure of both groups of FMT recipient mice between weeks 1–2 and weeks 3–4 post-FMT (Figure 2(c)). Overall, the gut microbiota of the recipient mice exhibited significant alterations in diversity, richness, and community structure at week 3 post-FMT, gradually resembling the donor profile by week 4.

By assessing the colonization time of the human-origin microbiota in the gut of HCC recipient mice, we found that the microbial abundance and structure in recipient mice in the imHCC group underwent significant changes in week 3 post-FMT and became stable in week 4, resembling the structure of the donor’s microbiota (Figure 2(d)). In contrast, recipient mice in the nmHCC group only achieved comparable microbial abundance to the donor at week 4 (Figure 2(e)). Therefore, the gut microbiota of patients with imHCC exhibited a more rapid colonization efficacy than that of patients with nmHCC, the result of which was further supported by the statistical analysis of microbial genus abundance (Figure S3). The functional dynamic changes in the gut microbiota of recipient mice after FMT were assessed. By week 4 post-FMT, the overall gene functions in recipient mice approached those in donors with HCC (Figure 2(f-g)). In summary, the gut microbiota of patients with HCC can colonize the intestinal tracts of HCC recipient mice. After four consecutive weeks of human-to-mouse FMT, a human flora-associated (HFA) intrahepatic metastasis HCC mouse model was successfully established.

### 2.3. Colonization of dysregulated gut microbiota promotes intrahepatic metastasis in the HCC mouse model and its potential underlying mechanisms

**Figure 3.**
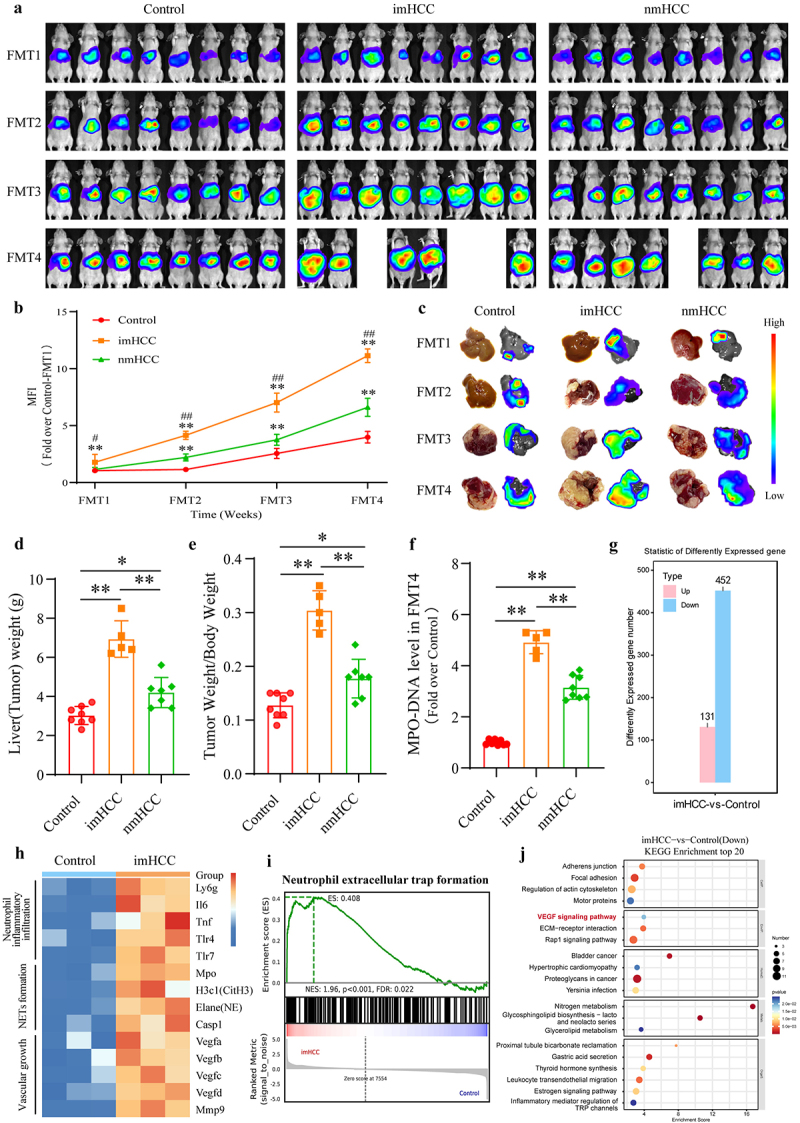
Colonization of dysregulated gut microbiota promotes intrahepatic metastasis in the HCC mouse model and its potential underlying mechanisms. (a). in *vivo* bioluminescence imaging of the growth and intrahepatic metastasis of tumors in mice during FMT. (b). Bioluminescence quantification analysis of tumors inside mice. (c). Dynamic changes in representative images of intrahepatic metastatic tumors and bioluminescence imaging after FMT. (d). Liver(Tumor) weight of mice from each group at week 4 post-FMT. (e). Ratio of Liver(Tumor) weight/body weight of mice from each group at week 4 post-FMT. (f). Levels of MPO-DNA in serum samples of mice from each group at week 4 post-fmt. (g). Different genes number between control and imHCC group. (h). Different genes enriched in neutrophil inflammatory infiltration, NETs formation and vascular growth pathways between control and imHCC group. (i). GSEA snapshots of neutrophil extracellular traps formation pathway enrichment analysis between control and imHCC group. (j). KEGG enrichment analysis of top 20 down-regulated pathways between control and imHCC group. The above animal model was constructed by MHCC97H Luc1 cells. HCC: hepatocellular carcinoma; imHCC: intrahepatic metastatic HCC; nmHCC: non-metastatic HCC; NETs: neutrophil extracellular traps; MPO: myeloperoxidase; FMT: fecal microbiota transplantation; VEGF: vascular endothelial growth factor; FMT1–4 represents the transplantation time for each week; MFI: mean fluorescence intensity. *p < 0.05, **p < 0.01 compared to the control group; and ^#^*p* < 0.05, ^##^*p* < 0.01 compared to the imHCC group.

We investigated the impact of the gut microbiota on tumor formation and intrahepatic metastasis by establishing mouse models bearing human liver cancer cell lines MHCC97H Luc1 and Huh-7 Luc1. To directly observe intrahepatic metastasis in HCC, one mouse was randomly selected and dissected each week. In *vivo* bioluminescence imaging indicated greater invasive and metastatic potential of MHCC97H cells, compared to Huh-7 cells. In the first week intrahepatic metastases were observed in each group of mice with FMT administration. Notably, the imHCC group exhibited significantly higher levels of metastasis compared to the Control and nmHCC groups. During the subsequent 2–4 weeks post-FMT, the imHCC group exhibited a rapid trend of intrahepatic metastasis. Notably, intrahepatic tumor metastasis and growth were particularly pronounced in weeks 3–4 post-FMT. The growth of intrahepatic metastatic tumors gradually reduced according to the seqeunce of imHCC group, nmHCC group and Control group (Figure 3(a-b)). Weekly liver dissection images also demonstrate that the growth of intrahepatic metastatic tumors in the imHCC group is significantly greater than that of the Control and nmHCC groups (Figure 3(c)). A similar trend was observed in the Huh-7 cells bearing mice. The invasive and metastatic potential of Huh-7 cells was lower than that of MHCC97H cells, with no significant metastasis detected during the first two weeks of post-FMT. From the third week onward, tumors in the imHCC group showed marked growth and intrahepatic invasion and metastasis compared to the Control and nmHCC groups. This trend remained consistent through the fourth week of post-FMT. While the nmHCC group exhibited higher tumor growth compared to the control group, the extent of intrahepatic invasion and metastasis was significantly lower than that observed in the imHCC group (Figure S4(a-c)). In week 4 post-FMT, mice in the imHCC and nmHCC groups exhibited a substantial decrease in body weight compared to those in the control group (Figure S4(d)). The liver (tumor) weight and tumor burden (tumor weight/body weight ratio) in the imHCC group were significantly higher than those in the Control and nmHCC groups (Figure 3(d-e) and S4(e)). Some mice experienced weight loss exceeding 20%, and even mortality. As the animals reached ethical endpoints, the experiment was terminated in the fourth week post-FMT. In addition, the study excluded the potential influence of ABX-induced depletion of intestinal commensal bacteria in mice (Figure S5). Therefore, in weeks 3–4 post-FMT, the colonization of the dysbiotic gut microbiota from patients with imHCC promoted intrahepatic metastasis of HCC mice.

By further investigating the underlying mechanisms, we found significant increase of the NETs formation marker, MPO-DNA, in the serum of the imHCC group compared to that of the control and nmHCC groups, consistent with the characteristics of patients with imHCC (Figure 3(f)). In addition, RNA sequencing results revealed that, compared to the Control group, the intrahepatic metastatic tumor tissues of mice in the imHCC group exhibited significant upregulation of genes associated with neutrophil inflammatory activation, NETs formation, and angiogenesis (Figure 3(h)). GSEA analysis indicated an upregulation of neutrophil extracellular trap formation in the imHCC group (Figure 3(i)). Meanwhile, KEGG enrichment analysis revealed a significant upregulation of VEGF signaling pathway (Figure 3(j)). Studies have shown that excessive formation of NETs can promote tumor angiogenesis.^19^ Therefore, the gut microbiota disequilibrium may contribute to HCC intrahepatic metastasis by promoting NETs formation.

### 2.4. Analysis of the microbial strains in the colonized gut microbiota that facilitate intrahepatic metastasis of HCC

**Figure 4.**
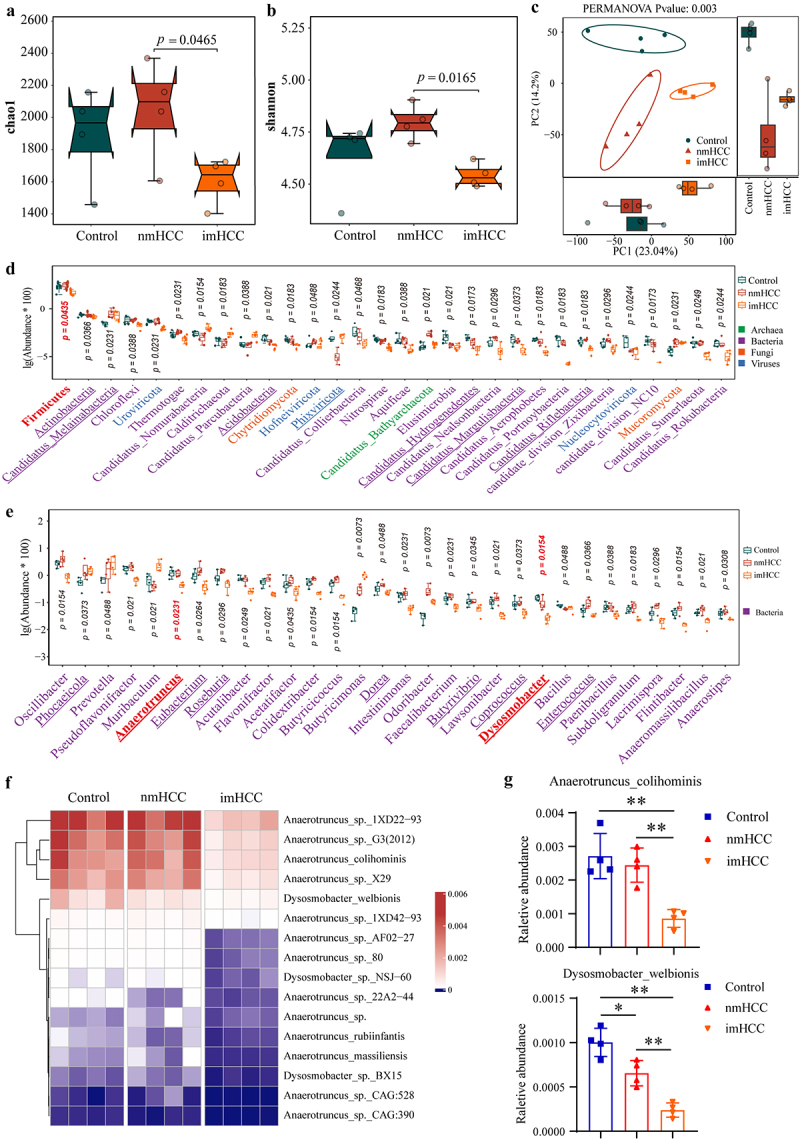
Analysis of the microbial strains within colonized gut microbiota that facilitate intrahepatic metastasis of HCC. (a) and (b). Analysis of the Chao1 index (a) and Shannon index (b) of gut microbiota in different groups; (c). PCA of gut microbiota in different groups; (d) and (e). Top 30 differential microorganisms at the phylum (d) and genus (e) levels in mice. (f). Heat map of the relative abundance of all species of *Anaerotruncus* and *Dysosmobacter* in mice. (g). Differential analysis of the relative abundance of *Anaerotruncus colihominis* and *Dysosmobacter welbionis* in mice. nmHCC group: mice transplanted with gut microbiota from patients with non-metastatic HCC; imHCC group: mice transplanted with gut microbiota from patients with intrahepatic metastatic HCC. *p < 0.05; **p < 0.01.

We analyzed the differences in gut microbiota among the groups of mice at the fourth week post-FMT. Compared to the control and nmHCC groups, the colonization of gut microbiota from patients with intrahepatic metastatic HCC led to a significant reduction in richness and diversity of the gut microbiota of mice in the imHCC group (Figure 4(a,b)). Based on PCA, microbial distribution in the imHCC group differed significantly between the control and nmHCC groups (Figure 4(c)). At the phylum and genus levels, among the top 30 differentially abundant microbial taxa, the relative abundance of (87.67%, 26/30) of phyla and 87.67% (26/30) of genera significantly decreased in the imHCC group compared to those of the control and nmHCC groups; this change was predominantly driven by alterations in bacterial composition. Among these changes, the levels of nine beneficial genera, including *Anaerotruncus* and *Dysosmobacter*, consistently decreased, which were observed in patients with intrahepatic metastatic HCC. Therefore, these alterations may originate from the colonization of the gut microbiota in patients with intrahepatic metastatic HCC (Figure 4(d-e)). Compared to the control and nmHCC groups, the relative abundance of all species within the *Anaerotruncus* and *Dysosmobacter* genera significantly decreased in imHCC mice (Figure 4(f)). In particular, *Anaerotruncus colihominis* and *Dysosmobacter welbionis* exhibited significant differences, consistent with the characteristics observed in patients with intrahepatic metastatic HCC (Figure 4(g)). These findings indicate that colonization of the gut microbiota in patients with intrahepatic metastatic HCC leads to disequilibrium in the HFA-intrahepatic metastatic HCC mouse model. The significant reduction in the abundance of the beneficial gut bacteria, *Anaerotruncus colihominis* and *Dysosmobacter welbionis*, was identified as a critical factor contributing to the intrahepatic metastasis of HCC.

### 2.5. Gut microbiota disequilibrium overstimulates NETs formation leading to tumour angiogenesis and necrosis

**Figure 5.**
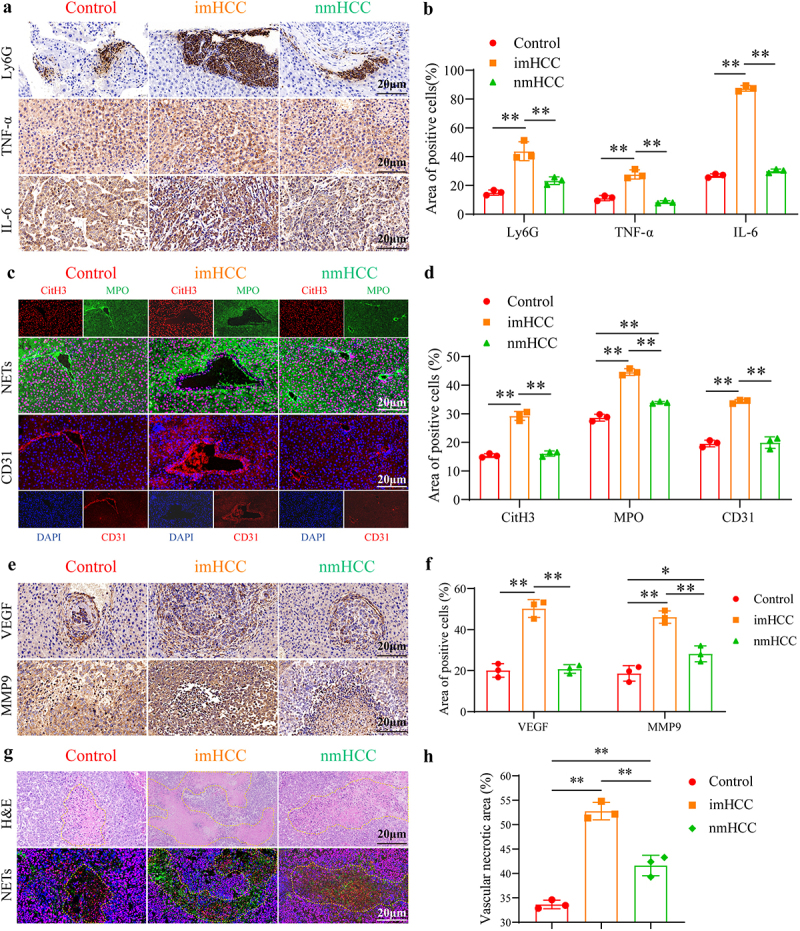
Gut microbiota disequilibrium overstimulates NETs formation leading to tumor angiogenesis and necrosis. (a) and (b). IHC staining analysis of the protein expression of Ly6G, TNF-α, and IL-6 in tumor tissues of mice; (c) and (d). If staining detection of the protein expression of the NETs formation markers (CitH3 and MPO) and CD31 in tumor tissues of mice; (e) and (f). IHC staining analysis of the protein expression of VEGF and MMP9 in tumor tissues of mice. (g) and (h). H&E staining of necrotic areas in the tumor tissues of mice, and if staining of the NETs formation markers (CitH3 and MPO). NETs: neutrophil extracellular traps, Ly6G: lymphocyte antigen 6 complex, locus G; tnf-α: tumour necrosis factor-α; IL-6: interleukin 6; CitH3: citrullinated histone H3; MPO: Myeloperoxidase; VEGF: vascular endothelial growth factor; MMP9: matrix metalloproteinase-9; IHC staining: immunohistochemical staining; if staining: immunofluorescence staining. *p < 0.05; **p < 0.01.

We investigated the effects of the gut microbiota disequilibrium on the inflammatory activation status of neutrophils and NETs formation in mouse tumors. Compared to the control and nmHCC groups, the expression levels of the neutrophil infiltration marker, Ly6G, and the inflammatory factors, TNF-α and IL-6, significantly increased in the imHCC group (Figure 5(a-b) and S6(a-b)). Simultaneously, the NETs formation markers, CitH3 and MPO, were significantly upregulated in the imHCC group. Western Blot analysis of the NETs formation markers (CitH3/MPO/NE) supported these findings (Figure 5(c-d) and S6(c-d)). Overall, the dysregulated gut microbiota in patients with intrahepatic metastasis of HCC induces inflammatory activation of neutrophils in tumor tissues, leading to excessive NETs formation. Excessive stimulation of NETs can promote tumor vascular growth.^11^ Fluorescence images indicate synchrony between the vascular formation marker, CD31, and NETs formation. NETs are also expressed synchronously in regions of active vascular growth in tumor tissues. Compared to the control and nmHCC groups, the imHCC group had a significant increase in the co-formation of NETs and CD31 (Figure 5(c-d)). In addition, the expression levels of the vascular growth factors, VEGF and MMP9, were significantly elevated in the imHCC group compared to those in the control and nmHCC groups (Figure 5(e-f) and S6(e-f)). This result suggests that excessive stimulation of NETs causes rapid neovascularization, which creates conditions for intrahepatic metastasis of HCC. The rapid growth of tumors is often accompanied by necrosis of the tumor tissues. The shedding of necrotic foci into the blood vessels can promote tumor metastasis.^20^ In this study, we found similarly active NETs formation in the necrotic foci region of tumor tissue. Moreover, compared to the control and nmHCC groups, the imHCC group displayed more necrotic areas and larger NETs formation areas in metastatic tumors (Figure 5(g-h)). To elucidate the role of NETs formation in intrahepatic metastasis of HCC, DNase I was used to clear NETs before intrahepatic metastasis in the HCC mouse model (Figure S7(a)). As expected, DNase I significantly inhibited the inflammatory activation of neutrophils (Figure S7(b-c) and S6(a-b)) and the formation of NETs (Figure S7(d-e) and S6(c-d)), which resulted in significant suppression of tumor vascular growth (Figure S7(f-g) and S6(e-f)), tissue necrosis (Figure S7(h-i)), and intrahepatic metastasis (Figure S4(j-p) and S8(a-d)). In summary, dysregulated gut microbiota dysbiosis from patients with intrahepatic metastatic HCC induces excessive NETs formation by stimulating the activation of neutrophilic inflammation. This process promotes tumor angiogenesis and tissue necrosis, ultimately leading to intrahepatic metastasis of HCC mice.

### 2.6. Healthy FMT can inhibit intrahepatic metastasis of HCC by reversing the gut microbiota disequilibrium

**Figure 6.**
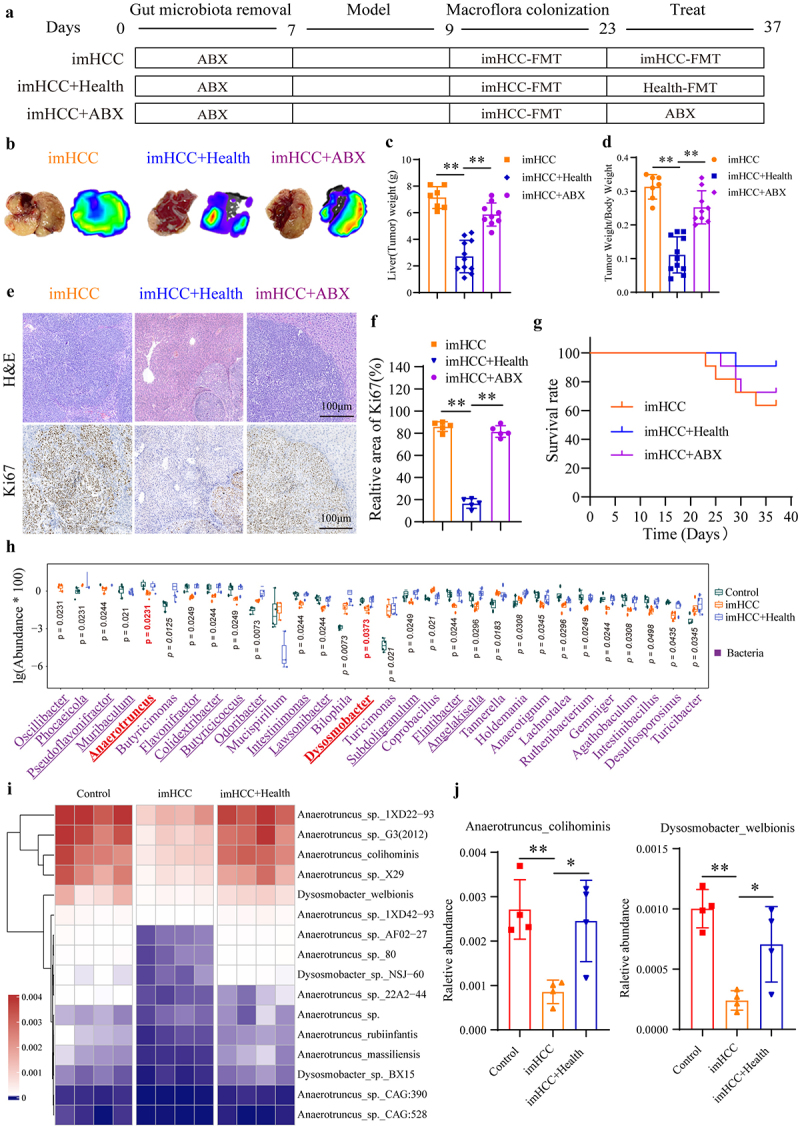
Healthy FMT can inhibit intrahepatic metastasis of HCC by reversing the gut microbiota disequilibrium. (a). Experimental design workflow. (b). Representative images of intrahepatic metastatic tumors and bioluminescence imaging. (c). Liver(Tumor) weight of mice from each group. (d). Ratio of Liver(Tumor) weight/Body weight of mice from each group. (e). Representative images of H&E staining and Ki67 IHC staining in each group of mice. (f). Extent of Ki67 positive expression in each group of mice. (g). Survival curves for each group of mice. (h). Top 30 differentially abundant microbial taxa at the genus level; (i). Heatmap depicting the relative abundance of all species of *Anaerotruncus* and *Dysosmobacter* of mice in each group; (j). Differences in the abundance of *Anaerotruncus colihominis* and *Dysosmobacter welbionis* in each group of mice. HCC: hepatocellular carcinoma; imHCC: intrahepatic metastatic HCC; nmHCC: non-metastatic HCC; FMT: fecal microbiota transplantation. *p < 0.05; **p < 0.01.

We explored a therapeutic strategy against metastasis in the HFA-HCC mouse model by adopting healthy FMT. The imHCC group was used to simulate the persistently dysbiotic gut microbiota environment observed in patients with intrahepatic metastatic HCC. The imHCC+Health group modeled the intervention of healthy FMT in HCC patients before the onset of intrahepatic metastasis. The imHCC+ABX group served as a negative control, simulating the depletion of gut microbiota through antibiotic treatment (Figure 6(a)). Compared to the imHCC group, the imHCC+Health group exhibited a significant reduction in intrahepatic metastatic tumors (Figure 6(b)). Furthermore, the Liver (Tumor) weight and tumor burden (Tumor weight/Body weight ratios) (Figure 6(c-d)), and the area and malignancy of the intrahepatic metastatic tumors (Figure 6e-f) were significantly reduced. The imHCC+Health group showed significantly increased survival rates and prolonged survival (Figure 6(g)). Consistent results were obtained when comparing the imHCC+PBS group with the imHCC+Health group (Figure S8(j-m)). However, ABX treatment did not have a significant effect. Additionally, consistent therapeutic effects were achieved in the Huh-7 cells-bearing animal model (Figure S8(e-i)). These results indicate that healthy FMT can effectively enhance the survival time and rate of mice by inhibiting intrahepatic metastasis and reducing the malignancy and burden of tumors. Based on joint analysis, the imHCC+Health group was found to have significantly higher richness (Chao1 index) and diversity (Shannon index) of gut microbiota than the control and imHCC groups (Figure S9(a-b)). The PCA results revealed distinct differences in microbial community structures among the three groups (Figure S9(c)). Further analysis at the phylum and genus levels revealed a significant increase in the abundance of 56.67% (17/30) of phyla (Figure S5(d)) and 80.0% (24/30) of genera (Figure 6(h)), which reversed to levels comparable to those in the control group. Notably, a substantial increase in beneficial bacteria, such as *Anaerotruncus colihominis* and *Dysosmobacter welbionis*, was identified as a crucial factor contributing to the intrahepatic metastasis of HCC (Figure 6(i-j)). In summary, healthy FMT effectively ameliorated disequilibrium in the gut microbiota of mice with HFA-intrahepatic metastatic HCC by increasing the number of beneficial bacteria, particularly *Anaerotruncus colihominis* and *Dysosmobacter welbionis*, thereby suppressing intrahepatic HCC metastasis.

### 2.7. Healthy FMT inhibits intrahepatic metastasis of HCC by regulating the excessive activation of NETs

**Figure 7.**
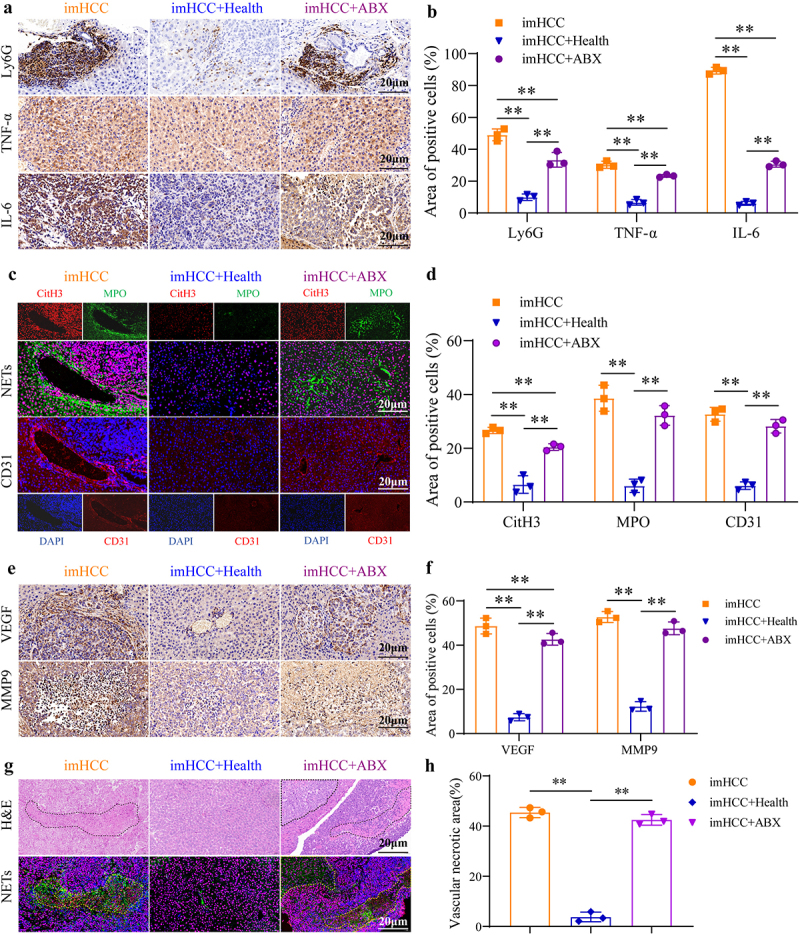
Healthy FMT inhibits intrahepatic metastasis of HCC by regulating the excessive activation of NETs. (a) and (b). Protein expression of Ly6G, TNF-α, and IL-6 in liver metastatic tumors of mice from each group; (c) and (d). IHC staining of NETs formation markers (CitH3 and MPO) and CD31 in liver metastatic tumors of mice from each group; (e) and (f). Protein expression of the tumor angiogenesis factors, MMP9, VEGF, and CD31, in liver metastatic tumors of mice from each group; (g) and (h). H&E staining of tissue necrotic areas in liver metastatic tumors of mice, and if staining of NETs formation markers (CitH3 and MPO). NETs: neutrophil extracellular traps, Ly6G: lymphocyte antigen 6 complex, locus G; TNF-α: tumour necrosis factor-α; IL-6: interleukin 6; CitH3: citrullinated histone H3; MPO: Myeloperoxidase; VEGF: vascular endothelial growth factor; MMP9: matrix metalloproteinase-9; IHC staining: immunohistochemical staining; if staining: immunofluorescence staining. *p < 0.05; **p < 0.01.

This study further elucidated the mechanism of healthy FMT for inhibiting intrahepatic metastasis of HCC. The levels of the neutrophil inflammatory infiltration marker, Ly6G, and inflammatory factors, TNF-α and IL-6, in tumor tissues significantly decreased in the imHCC+Health group and imHCC+ABX group, compared to the imHCC group (Figure 7(a-b)). Furthermore, NETs formation induced a significant decrease in the expression of MPO and CitH3 in the imHCC+Health group, compared to that in the imHCC group. However, no significant difference was found relative to the imHCC+ABX group (Figure 7(c-d)). This finding suggests that healthy FMT effectively inhibited gut microbiota disequilibrium-induced neutrophilic inflammatory activation and excessive NETs stimulation. Although ABX can suppress neutrophilic infiltration, no effect was found for excessive NETs formation. The above-mentioned study revealed that excessive NETs stimulation promotes tumor vascular growth. After undergoing healthy FMT, a significant reduction in NETs and CD31 co-formation was observed in the imHCC+Health group compared with the imHCC group. Furthermore, the levels of the vascular growth factors, VEGF and MMP9, were significantly decreased. No significant change was found in the imHCC+ABX group. These findings suggest that healthy FMT inhibits tumor tissue vascular growth by suppressing excessive NETs stimulation (Figure 7(e-f)). Correspondingly, necrosis of tumor tissue caused by excessive stimulation of NETs was significantly attenuated by healthy FMT (Figure 7(g-h)). Western blot analysis confirmed the inhibitory effect of healthy FMT on neutrophil inflammatory activation (Ly6G, TNF-α, and IL-6), NETs formation (CitH3, MPO and NE), and angiogenesis (CD31, MMP9, and VEGF) (Figure S6). Overall, healthy FMT controls the excessive stimulation of NETs by improving gut microbiota disequilibrium, thereby inhibiting tumor vascular growth and tissue necrosis and exerting an anti-intrahepatic metastasis effect in HFA-HCC mice. Although the clearance of dysbiotic gut microbiota by ABX can alleviate inflammatory infiltration in tumor tissues, this antibiotic does not effectively inhibit the progression of HCC.

## 3. Discussion

The presence of multiple intrahepatic metastases is a characteristic of HCC and serves as a significant risk factor for patient mortality.^4,21,22^ In this study, we firstly demonstrated that the gut microbiota disequilibrium could promote intrahepatic metastasis in mice with HCC. Similarly, clinical observations have indicated a dysregulated state of the gut microbiota in patients with intrahepatic metastatic HCC. Compared with healthy individuals and patients with non-metastatic HCC, a significant decrease in the diversity and richness of the gut microbiota, especially in the abundance of several core beneficial genera, was found in patients with metastatic HCC. Within this context, a significant decrease was observed in all species belonging to *Anaerotruncus* and *Dysosmobacter* genera, particularly *Anaerotruncus colihominis* and *Dysosmobacter welbionis*, which exhibited the most pronounced alterations. *Anaerotruncus colihominis*, a butyrate-producing probiotic, can regulate host immunity and promote disease recovery.^23^ *Dysosmobacter welbionis*, a newly isolated butyrate-producing bacterium from the human intestine, can modulate host metabolism and improve tissue inflammation.^24^ Therefore, dysregulation of the gut microbiota in patients with intrahepatic metastatic HCC may play a crucial role in the pathogenesis of such metastasis.

To validate this hypothesis, we established an HFA-intrahepatic metastatic HCC mouse model that sets a pioneering standard for gut microbiota research and its association with HCC, thereby providing an essential model for subsequent preclinical studies. Interestingly, transplantation of the gut microbiota from patients with intrahepatic metastatic HCC led to disequilibrium of the mouse gut microbiota. The richness and diversity of the mouse gut microbiota significantly decreased, and the abundance of various beneficial bacteria decreased, particularly for *Anaerotruncus colihominis* and *Dysosmobacter welbionis*. Notably, colonization of the dysregulated gut microbiota accelerated disease progression by promoting intrahepatic metastasis in HFA-HCC mice. This result confirms the pivotal role of dysbiotic gut microbiota in promoting the intrahepatic metastasis of HCC, particularly owing to the decrease of beneficial *Anaerotruncus colihominis* and *Dysosmobacter welbionis*.

We proceeded to evaluate the molecular mechanisms underlying the dysbiosis of the gut microbiota in promoting intrahepatic metastasis of HCC. Based on our clinical observations regarding the neutrophil-to-lymphocyte (NLR) ratio, intrahepatic metastasis of HCC might be associated with alterations in neutrophil activity as neutrophils are mainly recruited to combat infection and regulate the inflammatory response.^25^ Extracellular DNA mesh structures released from neutrophils also play a crucial role in host defense.^11^ Tumors resembling “non-healing wounds” are characterized by a persistent and uncontrolled inflammatory microenvironment. Multiple studies have highlighted the abundance of NETs within tumor metastatic foci.^26^ Excessive formation of NETs promotes tumor progression and metastasis by exacerbating inflammation.^27–29^ Consistently, we found significant increases in the serum levels of NETs formation markers (MPO) in mice transplanted with the gut microbiota of patients with intrahepatic metastatic HCC. RNA sequencing results indicate that the NETs formation is a potential mechanism by which gut microbiota disequilibrium promotes intrahepatic metastasis of HCC. The study validated this mechanism through in vivo experiments, revealing a significant increase in neutrophil inflammatory activation and NETs formation in intrahepatic metastatic tumors of the imHCC group. These results indicate a close association between the intrahepatic metastasis of HCC and gut microbiota disequilibrium, which stimulates excessive NETs formation. Prior studies have indicated that excessive formation of NETs can promote tumor vascular growth.^19^ The rapid growth of tumors is often accompanied by necrosis of the tumor tissues. The tumor cells from necrotic foci are prone to detach and enter the vascular system, leading to the migration of tumor cells into normal tissues, ultimately resulting in tumor metastasis.^20^ Our findings confirmed that excessive stimulation of NETs promotes high expression of the vascular growth factors, CD31, VEGF, and MMP9. Moreover, regions with high NETs concentrations had more necrotic foci. The rapid growth of the tumor vasculature creates conditions conducive to the invasion and migration of cancer cells. Altogether, this study revealed that the dysregulated gut microbiota in patients with intrahepatic metastatic HCC stimulates neutrophil inflammatory activation, triggering excessive formation of NETs, which further promotes tumor vascular growth and tissue necrosis.

Therapeutic strategies to counteract intrahepatic HCC metastasis were explored. In recent years, numerous studies have suggested that healthy FMT holds immense potential as an innovative approach in cancer therapy.^30^ This strategy can be used to treat gastrointestinal tumors at an early stage.^30–32^ Healthy FMT therapy was found to effectively inhibit intrahepatic metastasis in mice with HCC, thereby slowing the progression of the disease. In terms of its mechanism of action, healthy FMT reverses the gut microbiota disequilibrium in HFA-intrahepatic metastatic HCC mice. In addition, healthy FMT improves the richness and diversity of the gut microbiota and significantly enhances the abundance of beneficial bacteria, such as *Anaerotruncus colihominis* and *Dysosmobacter welbionis*. Improvement in the intestinal environment correspondingly suppresses neutrophil inflammatory activation, excessive formation of NETs, tumor vascular growth, and tissue necrosis in intrahepatic metastatic tumors in mice. In contrast, ABX therapy did not exhibit significant therapeutic effects owing to its inability to reverse the disruption caused by the dysregulated gut microbiota within the tumor microenvironment. Overall, healthy FMT effectively suppresses HCC intrahepatic metastasis by improving dysregulated gut microbiota.

The present study revealed gut microbiota dysbiosis in patients with intrahepatic metastatic HCC. By successfully constructing an HFA-intrahepatic metastatic HCC mouse model, we confirmed that the gut microbiota disequilibrium is a pivotal factor in the development of intrahepatic metastasis of HCC. Notably, the abundance of the beneficial bacteria, *Anaerotruncus colihominis* and *Dysosmobacter welbionis*, which play crucial roles, was significantly reduced. Dysbiosis primarily induces HCC intrahepatic metastasis by promoting neutrophil inflammatory activation, excessive NETs formation, and the subsequent facilitation of tumor vascular growth and tissue necrosis. This cascade accelerates HCC progression. Healthy FMT therapy effectively suppresses these phenomena. This study is the first to elucidate the role and mechanisms through which dysbiotic gut microbiota facilitate intrahepatic metastasis of HCC. Overall, healthy FMT has remarkable potential as a novel therapeutic strategy for the prevention and treatment of intrahepatic metastasis of HCC. However, further clinical development and application of this approach are required.

## 4. Experimental section

### 4.1. Experimental reagents and cells

**Table:** 

REAGENT or RESOURCE	SOURCE	IDENTIFIER
Ly-6 G (E6Z1T) Rabbit mAb	Cell Signaling	87048
Anti-Neutrophil Elastase	Affinity	AF0010
Anti-Histone H3	Affinity	AF0863
MPO Polyclonal antibody	proteintech	22225–1-AP
TNF Alpha Polyclonal antibody	proteintech	17590–1-AP
KI67 Polyclonal antibody	proteintech	27309–1-AP
IL-6 Monoclonal antibody	proteintech	66146–1-Ig
CD31 Monoclonal antibody	proteintech	66065–1-Ig
Goat Anti-Rabbit Recombinant Secondary Antibody (H+L)	proteintech	RGAR001
Anti-MMP9 antibody	abcam	ab38898
VEGF Rabbit Monoclonal Antibody	Beyotime	AF1309
Myeloperoxidase antibody	Santa Cruz Biotechnology	sc -390,109
Anti-GAPDH antibody	abcam	Ab181602
Alexa Fluor 488-labeled Goat Anti-Rabbit IgG(H+L)	Beyotime	A0423
REAGENT or RESOURCE		
Fetal bovine serum (FBS)	Meisen	CTCC-002-001
Trypsin-EDTA	Meisen	CTCC-002-006
Huh-7 Luc1 cell	Meisen	CTCC-0363-Luc1
phosphate buffered saline（PBS）	Meisen	CTCC-002-005
Dulbecco’s Modified Eagle’s Medium (DMEM)	Meisen	CTCC-002-008
Penicillin-Streptomycin	Gibco	15140122
Vancomycn	Abiowell	AWH0504e
Ampicillin	Abiowell	AWH0051f
Metronidazole	Abiowell	AWH0463c
Neomyein Sulfate	Abiowell	AWH0468e
BCA Protein Assay Kit	Solarbio	PC0020
Hematoxylin-Eosin (HE) Stain Kit	Solarbio	G1120
Mounting Medium, antifading (with DAPI)	Solarbio	S2110
Aprotinin from bovine lung	Solarbio	A8260
RIPA buffer(high)	Solarbio	R0010
Paraformaldehyde,4%	Solarbio	P1110
NcmBlot Rapid Transfer Buffer(20X)	Biotech	WB4600
NcmBlot Blocking Buffer	Biotech	P30500
Protein pregel	ACE Biotechnology	ET15412Gel
ECL chemiluminescent substrate kit	Biosharp	BL520B
Absolute ethyl alcohol	KERMEL	A0110999
Xylene	KERMEL	C03DREGA09011025ME
Isopropanol	KERMEL	C03DREGS09010073IP

### 4.2. Participant recruitment and sample collection

From May 2018 to December 2022, fecal samples were collected from 54 patients with HCC (28 with intrahepatic metastatic HCC and 26 with non-metastatic HCC) at the First Affiliated Hospital of Hunan University of Chinese Medicine. Fecal samples from 30 healthy individuals were used as controls (Table S1). Each participant signed an informed consent form, and was diagnosed with HCC according to the guidelines for the diagnosis and treatment of primary liver cancer (2022 edition) issued by the National Health Commission. Dynamic enhanced CT scans, multiparameter MRI scans, and serum alpha-fetoprotein (AFP) examination were used for the diagnosis. The study utilized imaging characteristics to distinguish between intrahepatic metastatic HCC, multifocal HCC, and non-metastatic HCC (Figure S1). The study compared the general demographic and clinical variables of three cohorts, such as age, gender, BMI, tumor size, tumor number, tumor stage, metastasis status, extrahepatic diseases, neutrophil count, neutrophil-to-lymphocyte ratio (NLR), platelets, liver function indicators (total protein, albumin, globulin, transaminases, alkaline phosphatase, γ- Glutamine transpeptidase, total bilirubin, direct bilirubin, indirect bilirubin, total bile acids), liver function grade, serum AFP levels, and Barcelona Clinic Liver Cancer (BCLC) stage. Fecal samples were collected on the second morning of hospitalization and before treatment to eliminate the effect of hospitalization on the gut microbiota of patients. Fresh fecal samples from all participants were immediately frozen in liquid nitrogen and stored at −80°C. Peripheral blood was collected after overnight fasting and placed in anticoagulant tubes. This study was approved by the Ethics Committee of the First Affiliated Hospital of Hunan University of Chinese Medicine (approval no. HN-LL-KY-2022-020-02), following the principles outlined in the Declaration of Helsinki and local laws.

### 4.3. Preparation of faecal microbiota suspension

Fresh fecal samples were collected from patients with intrahepatic metastatic HCC (imHCC, *n* = 28) and those with non-metastatic HCC (nmHCC, *n* = 26) (Table S1). A fecal microbiota suspension was prepared immediately after the collection of fresh feces in sterile containers. A clean FMT protocol was employed for the preparation,^33–35^ which comprised microfiltration and centrifugation-enrichment methods, with repeated centrifugation and washing. Using this process, the fecal bacteria were enriched and purified within 1 h under aseptic conditions. The prepared fecal suspension had a microbial concentration of 2.5 × 10^12^/50 mL and was stored at −80°C until use. Before use, the donor fecal suspension was thawed and homogenized. Healthy FMT was conducted using the healthy fecal microbiota transplantation capsules provided by Shenzhen JunChangyi Co., Ltd (M20C0323MCJ). According to the *European Consensus Conference on Faecal Microbiota Transplantation in Clinical Practice*,^36^ the effectiveness and safety of health-FMT depend on two key factors: Donor Selection and Preparation of Fecal Material. In this study, we strictly adhered to the standards outlined in the consensus to minimize and prevent any adverse events associated with fecal material infusion. The main procedures included conducting medical interviews with potential FMT donors, performing comprehensive blood and stool testing, conducting additional interviews on the day of donation, and preparing fecal material using a frozen suspension method. These rigorous steps ensured the careful selection of healthy donors to facilitate the transplantation of a robust and healthy gut microbiota.

### 4.4. Animal experiment

Using fecal microbiota suspensions prepared from patients and healthy individuals, as described in section 4.3, a human-to-mouse FMT experiment was conducted. The BALB/c nude mice were divided into three groups: control group, administered PBS via gavage; imHCC group, administered fecal suspension from patients with imHCC via gavage; and nmHCC group, administered fecal suspension from patients with nmHCC via gavage. A total of 33 mouse models were constructed using MHCC97H Luc1 cells, with 11 mice per group. Additionally, 24 mouse models were constructed using Huh-7 Luc1 cells, with 8 mice per group. Each week, one mouse was randomly dissected to observe intrahepatic tumor metastasis. Before transplantation, the symbiotic gut microbiota of the two FMT recipient groups was cleared using ABX.^37,38^ Thereafter, an intrahepatic metastatic HCC tumor model was established. Two days after the wounds of mice recovered, human-mouse FMT was initiated. The doses of PBS and fecal suspension were 200 μL/day and were administered once daily for four consecutive weeks.^33–35^ Human gut microbiota colonisation in mice and the growth of intrahepatic metastatic tumors were monitored weekly.

Treatment experiment: After clearing the symbiotic gut microbiota with ABX, an intrahepatic metastatic HCC mouse model was constructed using BALB/c nude mice. Mice were divided into the following four groups: imHCC group, administered a fecal suspension from patients with imHCC for 4 weeks via gavage; imHCC+Health group, administered a fecal suspension from patients with imHCC for 2 weeks via gavage, and then treated with healthy FMT for 2 weeks; and imHCC+ABX group, administered a fecal suspension from patients with imHCC for 2 weeks via gavage, and then treated with the antibiotic, ABX, for 2 weeks. The imHCC+DNase I group was administered fecal suspension from patients with imHCC for 2 weeks via gavage, and then treated with DNase I for 2 weeks. During the subsequent 2 weeks of treatment in the imHCC+DNase I group, imHCC+Health group, and imHCC+ABX group, imHCC-FMT was performed every 3 days to stabilize the colonization of the gut microbiota from patients with intrahepatic metastatic HCC. A total of 44 mice were constructed using MHCC97H Luc1 cells (n = 11/group). Additionally, 24 mice were constructed using Huh-7 Luc1 cells (n = 6/group). The fecal suspension dose was 200μL/mouse/day. In addition, 100 U/mouse/day of DNase I was intraperitoneally administered.^37^ After the intervention, live animal imaging was performed to measure the fluorescent area of the tumours, and mouse feces were collected for metagenomic sequencing.

The growth status of all mice was monitored daily, and body weight was measured every 3 days. At the end of the intervention, the animal was exsanguinated via cervical dislocation. Mouse livers and spleens were weighed to assess the tumor burden, and serum and liver tumor tissues were collected for subsequent analyses. The research protocol was reviewed and approved by the Experimental Animal Welfare and Ethics Review Committee of Hunan University of Chinese Medicine (ethics approval number LLBH-202106090001).

### 4.4. Pre-clearance of endogenous microbiota before FMT

Prior to FMT, the endogenous microbiota of mice was eliminated following a protocol adapted from previous studies.^38^ An antibiotic cocktail (ABX) consisting of ampicillin (1 g/L), neomycin (1 g/L), metronidazole (1 g/L), and vancomycin (0.5 g/L), was prepared. ABX was added to the drinking water of mice designated for FMT at a dosage of 10 mg of antibiotic per mouse per day.^37^ Mice were continuously provided antibiotic-containing water for one week, with water changes conducted every three days. Collect fecal samples from mice before and after ABX intervention, and conduct metagenomic sequencing to assess the elimination of intestinal commensal bacteria in mice.^37^

### 4.5. Cell lines and culture conditions

The MHCC97H Luc1 and Huh-7 Luc1 cell line was obtained from Zhejiang Meisen Cell Technology Co., Ltd., and its identity was confirmed through short tandem repeat (STR) analysis to ensure the absence of contamination. The cells were cultured in complete medium supplemented with 10% heat-inactivated fetal bovine serum and 1% (v/v) penicillin-streptomycin, and maintained in a humidified atmosphere at 37°C with 5% CO_2_.

### 4.6. Preparation of HFA intrahepatic metastatic HCC mouse model

To establish the liver cancer metastasis model, 3 × 10^6^ MHCC97H Luc1 cells or Huh-7 Luc1 cells carrying the luciferase gene were injected into the spleen of BALB/c nude mice.^39^ Briefly, mice were anaesthetised with 100 mg/kg pentobarbital sodium via intraperitoneal injection. Sterile drapes and gloves were used to maintain aseptic conditions during the procedure. A 1.5 cm incision was made near the hind limbs of the lower abdomen. Using curved forceps, all underlying muscles were dissected and 3 × 10^6^ MHCC97H Luc1 cells or Huh-7 Luc1 cells were injected into the spleen. The abdominal incision was closed using 5–0 subcutaneous sutures (Vicryl). Postoperative care involved monitoring mice for signs of pain or distress four times per day for three days. FMT was initiated on the third day post-modeling, when the wounds in the mice had healed. Tumor volume was monitored weekly using in *vivo* bioluminescence imaging. The tumor volume (V, cm^3^) was calculated by dividing the luminescence intensity (photons/s) by the initial value.

### 4.8. Metagenomics sequencing

Microbial genomic DNA was extracted from samples using the QIAamp® Fast DNA Stool Mini Kit (Qiagen, Hilden, Germany). Library construction was performed using a TruSeq Nano DNA LT Sample Preparation Kit (Illumina, San Diego, CA, USA). Library construction, sequencing, and data analysis were performed by Shanghai Ouyi Biomedical Technology Co., Ltd. The libraries were sequenced on the Illumina NovaSeq 6000 sequencing platform, which generated 150 bp paired-end reads. To construct gene sets, CDHIT (v 4.5.7) was used to build non-redundant gene sets from predicted genes in all samples, with clustering parameters set at 95% identity and 90% coverage between sequences. The longest gene in each cluster set was selected as the representative sequence for that gene set. After obtaining representative gene sequences, bowtie2 (v 2.2.9) aligned clean reads of each sample to the non-redundant gene set (95% identity), and the abundance information of genes in the corresponding samples was calculated.

Taxonomic annotations were obtained using the NR library, and species abundance was calculated by integrating the gene abundance information corresponding to each species. Abundance profiles were constructed at various taxonomic levels, including the Domain, Kingdom, Phylum, Class, Order, Family, Genus, and Species. Representative gene sequences (amino acid sequences) were annotated against the NR library, KEGG, COG, SWISSPROT, GO, and other databases using the DIAMOND software (v 0.9.7), with a BLAST e-value threshold set at 1e-5. For carbohydrate-active enzyme (CAZy) annotation, gene sets were aligned with those in the CAZy database using the hmmscan tool (v 3.1) to obtain annotation information for carbohydrate-active enzymes. The abundance of genes corresponding to carbohydrate-active enzymes was summed to calculate the abundance of the respective carbohydrate-active enzymes. The R package (v 3.2.0) was used for PCA and plotting based on the taxonomic or functional abundance profiles. The NMDS distance matrices were calculated, and plots were generated for analysis. Differential analysis was performed using the T test/Wilcoxon, and LEfSe was employed for the differential analysis of taxonomic or functional abundance profiles.

### 4.9. Immunofluorescence staining

After fixation with 4% paraformaldehyde, mouse tissue specimens were embedded and sectioned at a thickness of 5 μm. Following deparaffinization, antigen retrieval was performed for 20 min, and the sections were washed three times with PBS for 5 min each. Subsequently, 3% H_2_O_2_ was applied at room temperature for 10 min, and three washes with PBS were performed for 5 min each to deactivate the endogenous enzymes. The sections were then treated with 0.3–0.5% TritonX-100 at room temperature for 10 min, washed three times with PBS for 5 min each before blocking with goat serum for 1 h, and incubated with the primary antibodies overnight at 4°C. After warming for 30 min, the sections were washed three times with PBS for 5 min each and then incubated with secondary antibodies in the dark at 37°C for 1 h. Subsequently, the sections were washed three times with PBS for 5 min each, stained with DAPI for 10 min, and washed three times with PBS for 5 min each. Finally, the slides were mounted using an anti-fade mounting medium. Fluorescent images were captured using a fluorescence microscope. ImageJ software was used for semi-quantitative analysis of three representative regions under the same settings.

### 4.10. Haematoxylin-eosin (H&E) staining

Paraffin-embedded sections were de-paraffinized in xylene for 15 min (repeated thrice) and dehydrated with 100% ethanol for 2 min, 95% ethanol for 2 min, 75% ethanol for 2 min, and distilled water for 2 min. The sections were stained with hematoxylin for 10 min, rinsed with tap water for 5 min, stained with eosin for 3 min, and rinsed with tap water for 5 min. Subsequently, sections were dehydrated with 100% ethanol and xylene for 30 s each. After dehydration and clarification, the sections were mounted using a neutral resin and photographed.

### 4.11. Immunohistochemistry staining

Immunohistochemical staining of the paraffin-embedded sections was performed using the biotin-streptavidin-peroxidase complex method. Briefly, after rehydration and microwave antigen retrieval, the sections were incubated with primary antibodies overnight at 4°C, followed by secondary antibodies (GeneTech) at 37°C for 30 min. Staining was performed using 3,3’-diaminobenzidine tetrahydrochloride, and counterstaining was conducted with Mayer’s hematoxylin. The immunohistochemical results were evaluated by two independent researchers. ImageJ software was used to analyze three representative regions under the same settings.

### 4.12. Measurement of MPO-DNA levels in mouse serum

The quantification of MPO-DNA complexes in mice serum was performed using MPO-DNA ELISA Kit from Shanghai Hengyuan Biotechnology Co., Ltd (Catalog No. HB1200-Mu). Briefly, serum samples were added to wells pre-coated with specific antibodies against MPO, followed by incubation to allow binding of MPO-DNA complexes. After washing to remove unbound components, a secondary antibody specific to DNA, conjugated with an enzyme, was added. The reaction was visualized using a chromogenic substrate, and the absorbance was measured at the appropriate wavelength using a microplate reader. Quantification was achieved by comparing the absorbance values to a standard curve generated with known concentrations of MPO-DNA complexes.

### 4.13. RNA sequencing analysis

Intrahepatic metastatic tumor tissues were collected from mice in the Control and imHCC groups following the establishment of the HFA-intrahepatic metastatic HCC mouse model. RNA was extracted from three biological replicates using the RNeasy kit (Qiagen China, Shanghai, China), and the quantity and integrity of the RNA were evaluated with the RNA Nano 6000 Assay Kit on the Bioanalyzer 2100 system (Agilent Technologies, CA, USA). Libraries were prepared following the manufacturer’s protocol and sequenced on the Illumina NovaSeq 6000 platform (Illumina, San Diego, CA, USA).

### 4.14. Statistical analysis

Statistical analyses were conducted using SPSS version 26.0. The Shapiro-Wilk test was performed to assess the normal distribution of the data. Normally distributed data are expressed as mean ± standard deviation (Mean ± SD), and non-normally distributed data are presented as the lower quartile, median, and upper quartile. For normally distributed data, one-way analysis of variance (ANOVA) was performed, followed by Tukey’s test for post-hoc pairwise comparisons unless stated otherwise in the figure legend. The Brown-Forsythe test was employed for unequal variances, and the Dunnett T3 test was used for multiple comparisons between groups. An independent sample t-test was conducted to compare two groups. Non-normally distributed data were analyzed using the Kruskal-Wallis non-parametric test, followed by the Mann-Whitney U test for pairwise comparisons between groups. Statistical significance was indicated by *p* < 0.05.

## Supplementary Material

Supplemental Material_Revised.docx

## Data Availability

All data are contained in the article and the supporting information. All Supplemental Material is deposited in the figshare (10.6084/m9.figshare.25854724). All additional datasets and materials are available from the corresponding author upon reasonable request.
