# Supplementary material for: Dysregulation of gut microbiota stimulates NETs-driven HCC intrahepatic metastasis: therapeutic implications of healthy faecal microbiota transplantation
Source: Gut Microbes. 2025 Mar 18;17(1):2476561. doi: 10.1080/19490976.2025.2476561 (PMC11925110; doi:10.1080/19490976.2025.2476561)
Supplement: Supplemental Material_Revised.docx [file KGMI_A_2476561_SM9088.docx]

**Supplemental Material**

**Table S1. Clinical Sample Collection and Participant Characteristics**


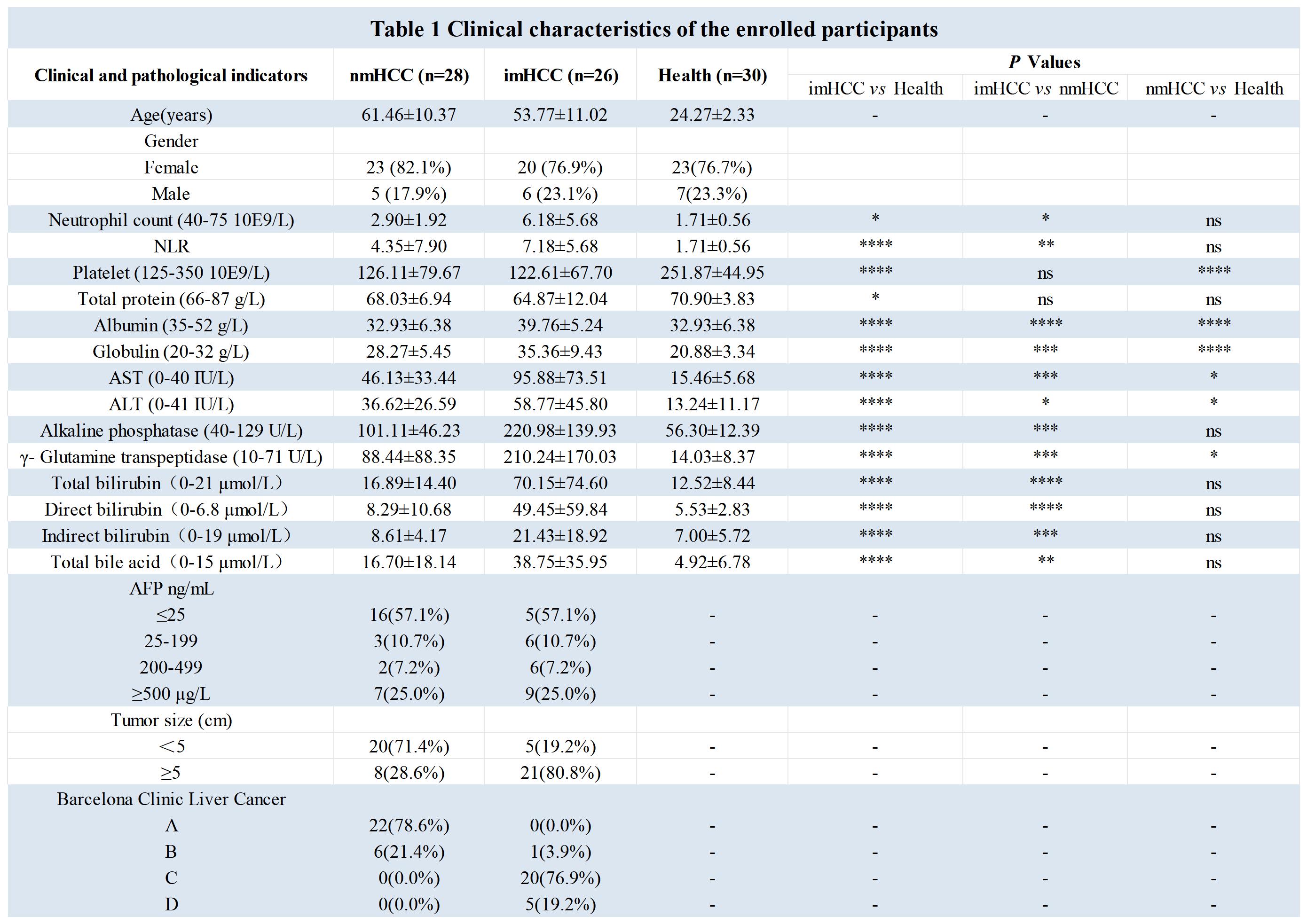


HCC: hepatocellular carcinoma; NLR: Neutrophil-to-lymphocyte ratio; imHCC: intrahepatic metastatic HCC; nmHCC: non-metastatic HCC; ALT: Alanine aminotransferase; AST: Aspartate aminotransferase; AFP: Alpha-FetoProtein. **P* <0.05; ***P* <0.01; ****P* <0.001; *****P* <0.0001; ns, not significant.

**Figure S1.** **Differentiating the characteristic imaging features of intrahepatic metastatic HCC, multifocal HCC, and non-metastatic HCC patients.**


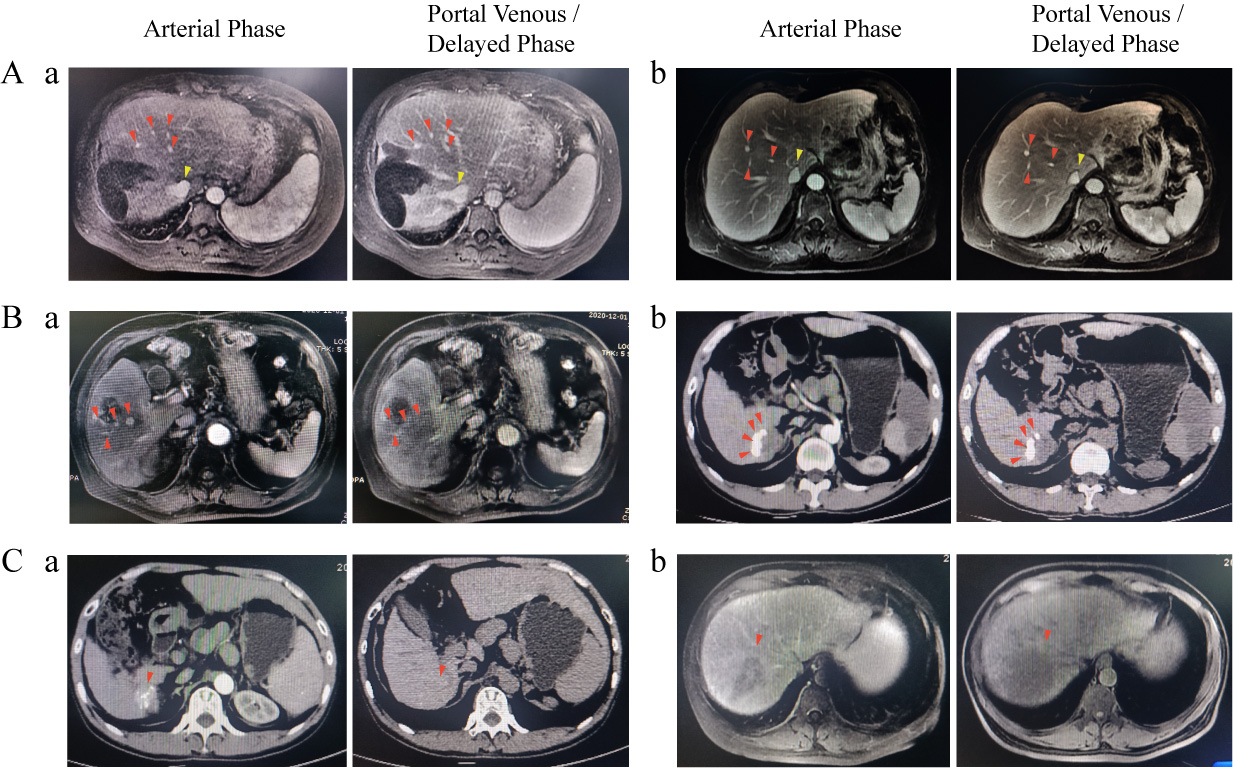


Figure S1. Differentiating the characteristic imaging features of intrahepatic metastatic HCC, multifocal HCC, and non-metastatic HCC patients.A. Representative imaging of intrahepatic metastatic HCC patients using unenhanced and contrast-enhanced MRI of the upper abdomen: a. Intrahepatic metastatic HCC in Patient 1; b. Intrahepatic metastatic HCC in Patient 2. The yellow arrows indicate the primary lesion, while the red arrows highlight its distal satellite lesions, exhibiting the characteristic "satellite sign." The lesions are relatively well-defined in shape. During the arterial phase, they demonstrate significant enhancement, remain hyperintense relative to normal liver parenchyma during the portal venous phase, and show reduced enhancement in the delayed phase, consistent with the "rapid wash-in and slow wash-out" pattern.B. Representative imaging of multifocal HCC patients using unenhanced and contrast-enhanced MRI of the upper abdomen: a. Multifocal HCC in Patient 1; b. Multifocal HCC in Patient 2. The red arrows indicate multiple independent lesions, distributed diffusely and varying in size. The tumor boundaries are poorly defined, with irregular shapes. On contrast-enhanced imaging, the lesions exhibit a "rapid wash-in and rapid wash-out" enhancement pattern. C. Representative imaging of non-intrahepatic metastatic HCC patients using unenhanced and contrast-enhanced MRI of the upper abdomen: a. Non-intrahepatic metastatic HCC in Patient 1; b. Non-intrahepatic metastatic HCC in Patient 2. The red arrows indicate the lesions. Both patients are classified as stage Ia or Ib, with no evidence of tumor thrombus, abdominal lymph node involvement, or distant metastasis.

**Figure S2. The administration of antibiotics ABX effectively eliminated the intestinal intestinal commensal bacteria in recipient mice before FMT**


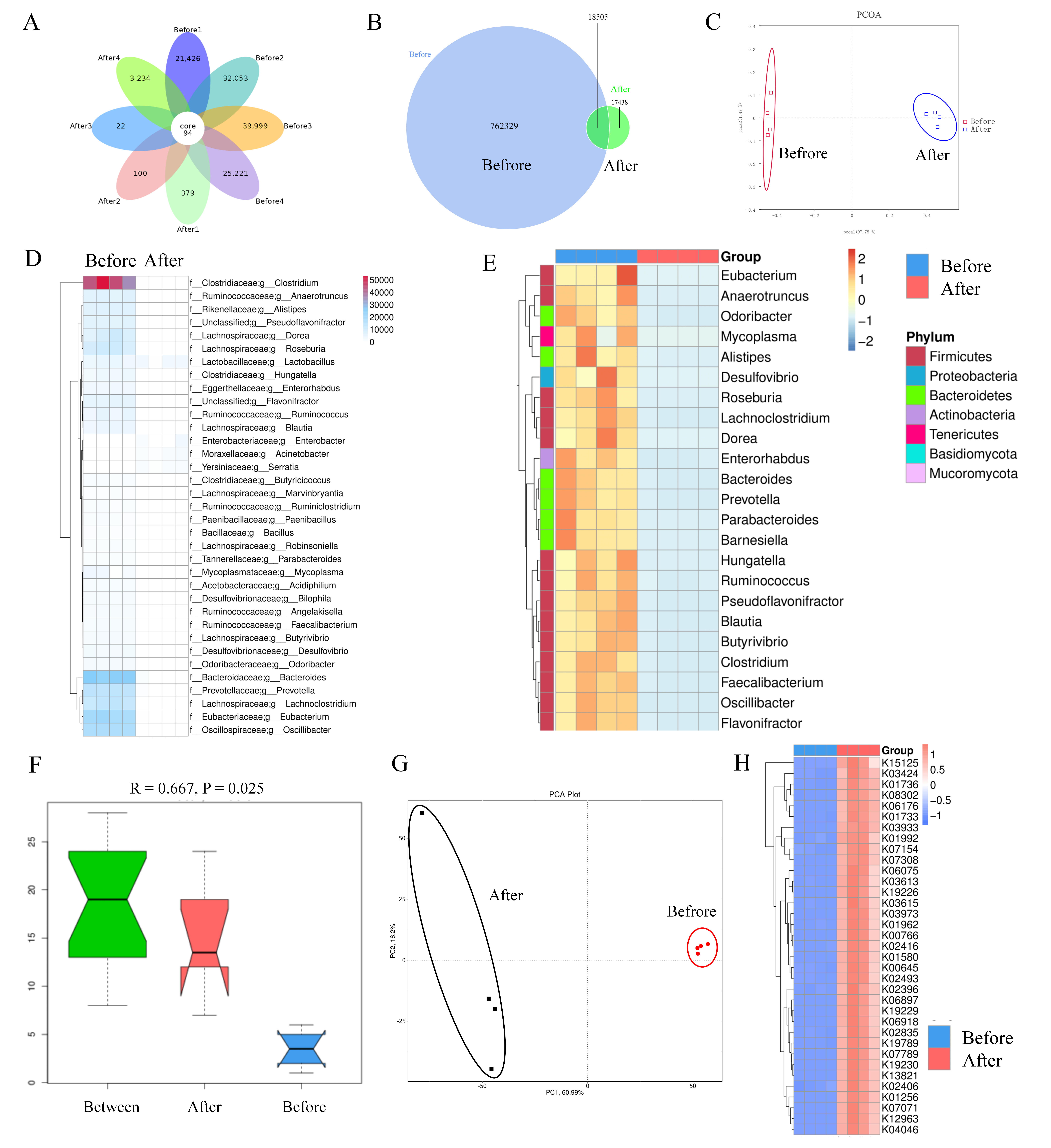


Figure S2. The administration of antibiotics ABX effectively eliminated the intestinal intestinal commensal bacteria in recipient mice before FMT. A-B. Petal and Venn diagrams illustrating the number of genes in the mouse gut microbiota before and after bowel cleansing before and after ABX treatment; C. PCoA analysis of the mouse gut microbiota before and after ABX treatment; D. Comparison of the number of genes in the mouse gut microbiota before and after ABX treatment; E. Comparison of the gene abundance in the mouse gut microbiota before and after ABX treatment; F. Anosim analysis of the functional abundance of the mouse gut microbiota before and after ABX treatment; G. PCA analysis of the gene function of the mouse gut microbiota before and after ABX treatment; H. Abundance of gene function in the mouse gut microbiota before and after ABX treatment.

**Figure S3. Dynamic changes in the relative abundance of human-derived donor gut microbiota in the gut of recipient mice after FMT.**


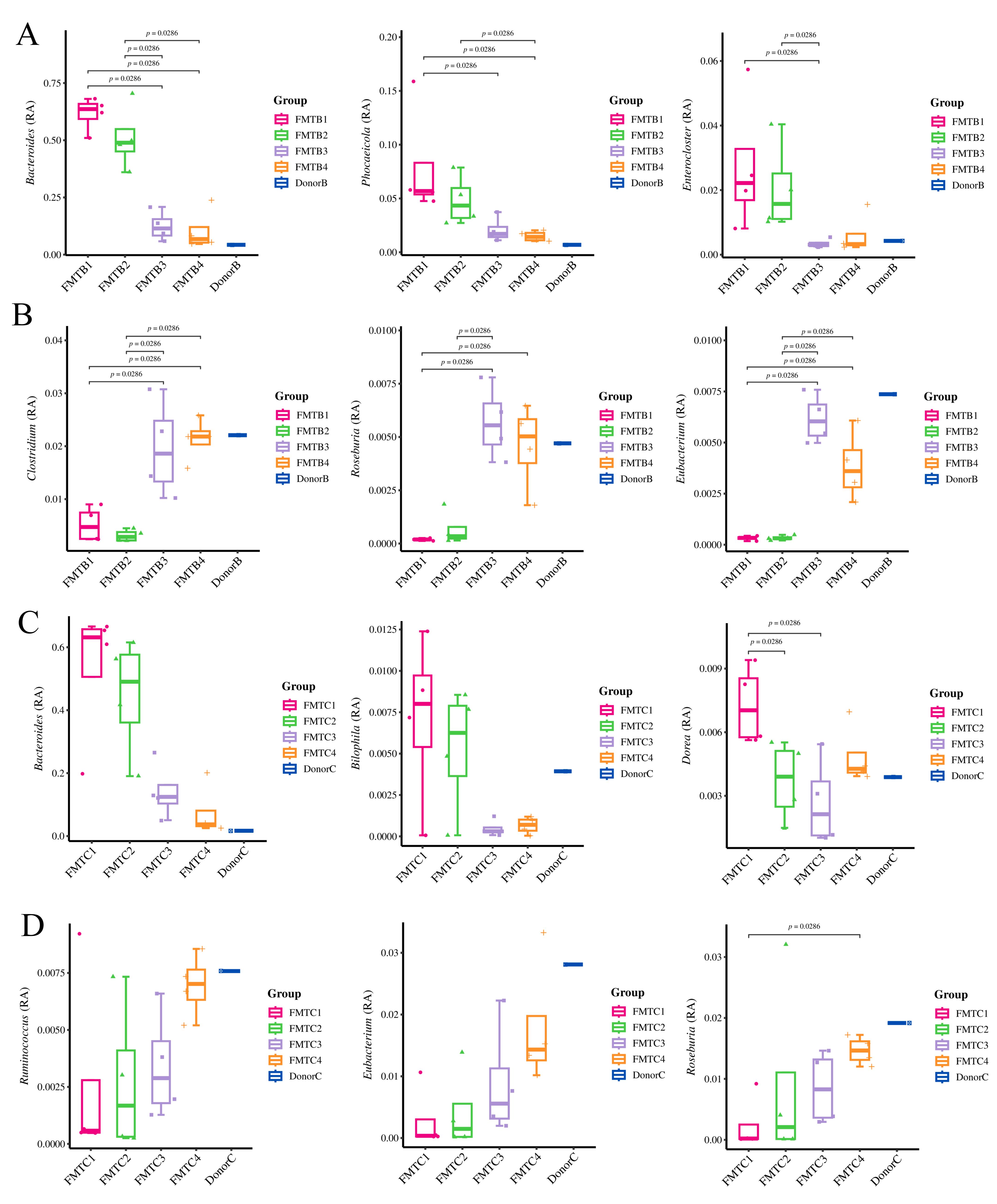


Figure S3. Dynamic changes in the relative abundance of human-derived donor gut microbiota in the gut of recipient mice after FMT. A-B. Dynamic changes in the relative abundance of characteristic microbial taxa from donor B in the gut of recipient mice post-FMT, depicting both decreases (A) and increases (B) compared to the donor. C-D. Dynamic changes in the relative abundance of characteristic microbial taxa from donor C in the gut of recipient mice post-FMT, illustrating both decreases (C) and increases (D) compared to the donor. Donor B: donors with imHCC (n=28); Donor C: donors with nmHCC (n=26). FMT1-4 represents the transplantation time for each week.

**Figure S4.** **Colonization of dysregulated gut microbiota promotes intrahepatic metastasis in the HCC mouse model.**


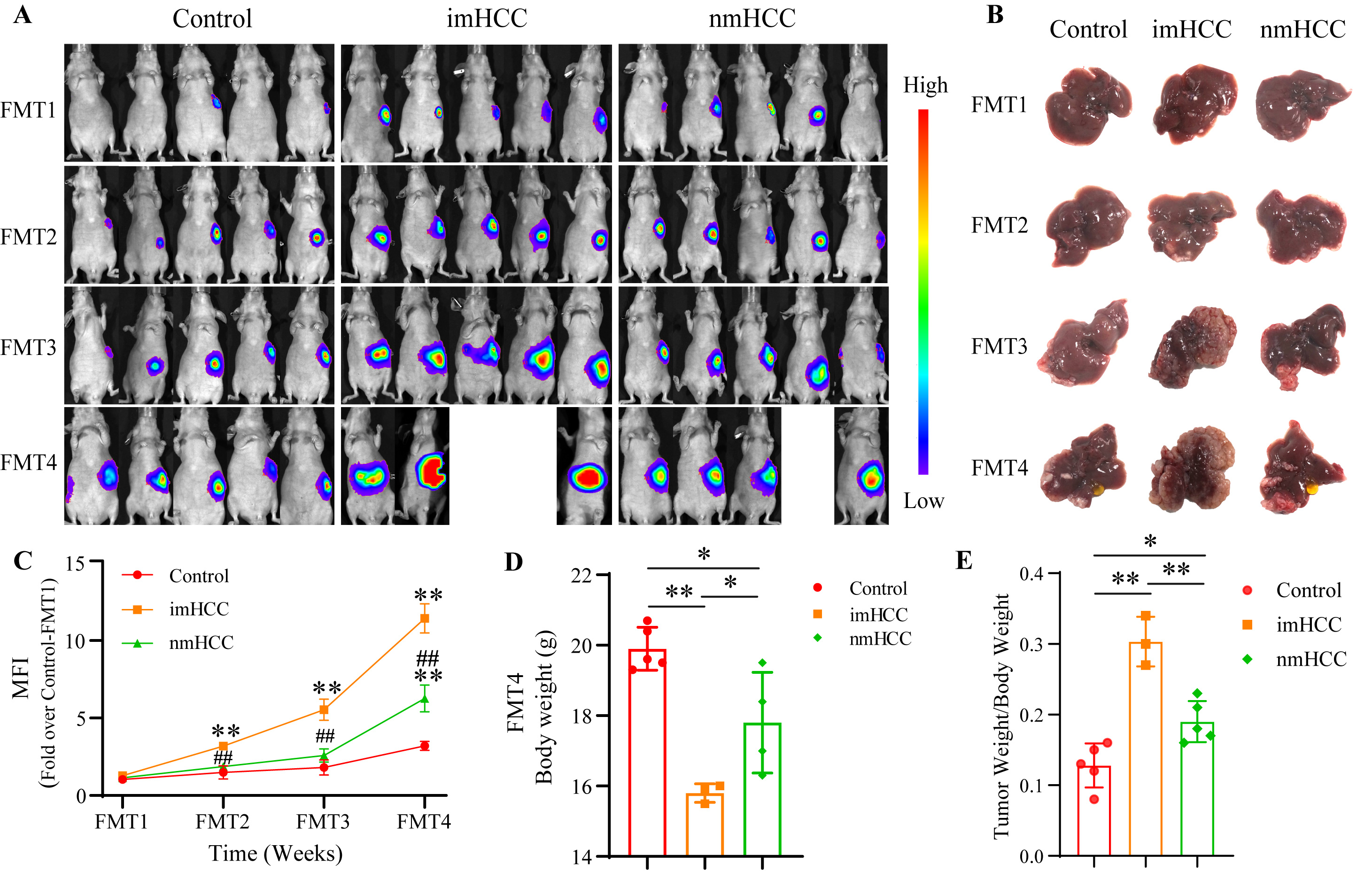


Figure S4. Colonization of dysregulated gut microbiota promotes intrahepatic metastasis of HCC mice. A. In vivo bioluminescence imaging of the growth and intrahepatic metastasis of tumours in mice during FMT. B. Dynamic changes in representative images of intrahepatic metastatic tumours after FMT. C. Bioluminescence quantification analysis of tumours inside mice. D. Body weight of mice from each group at week 4 post-FMT. E. Ratio of Liver(Tumor) weight/Body weight of mice from each group.at week 4 post-FMT. The above animal model was constructed by Huh-7 Luc1 cells. HCC: Hepatocellular carcinoma; imHCC: Intrahepatic metastatic HCC; nmHCC: Non-metastatic HCC; FMT: faecal microbiota transplantation; FMT1-4 represents the transplantation time for each week; MFI: Mean Fluorescence Intensity. ^*^*P* < 0.05, ^**^*P* < 0.01 compared to the Control group; and ^#^*P* < 0.05, ^##^*P* < 0.01 compared to the imHCC group.

**Figure S5. The depletion of gut commensal bacteria in mice by ABX had no impact on the experimental results.**


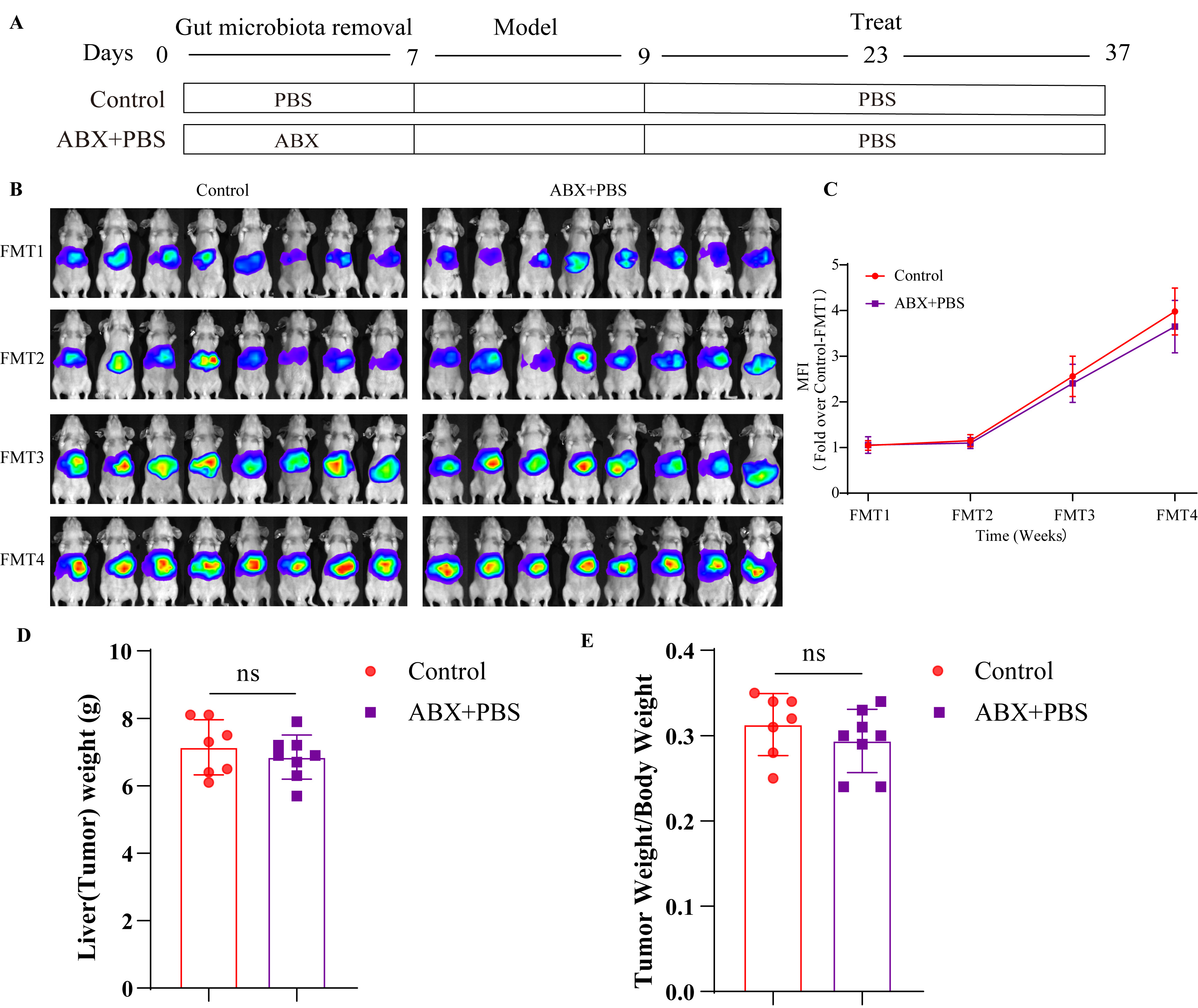


Figure S5. The depletion of gut commensal bacteria in mice by ABX had no impact on the experimental results. A. Experimental design workflow. B. In vivo bioluminescence imaging of the growth and intrahepatic metastasis of tumours in mice during FMT. C. Bioluminescence quantification analysis of tumours inside mice. D. Liver(Tumor) weight of mice between Control and ABX+PBS group at week 4 post-FMT. E. Ratio of Tumor weight/Body weight of mice Control and ABX+PBS group at week 4 post-FMT. The above animal model was constructed by MHCC97H Luc1 cells. HCC: Hepatocellular carcinoma; imHCC: Intrahepatic metastatic HCC; nmHCC: Non-metastatic HCC; FMT: faecal microbiota transplantation; FMT1-4 represents the transplantation time for each week; MFI: Mean Fluorescence Intensity. **P* <0.05; ***P* <0.01.

**Figure S6.** **Western blot was performed to detect the expression of neutrophil inflammatory activation markers, NETs formation markers, and vascular growth factors in the tumor tissues of each group of mice.**


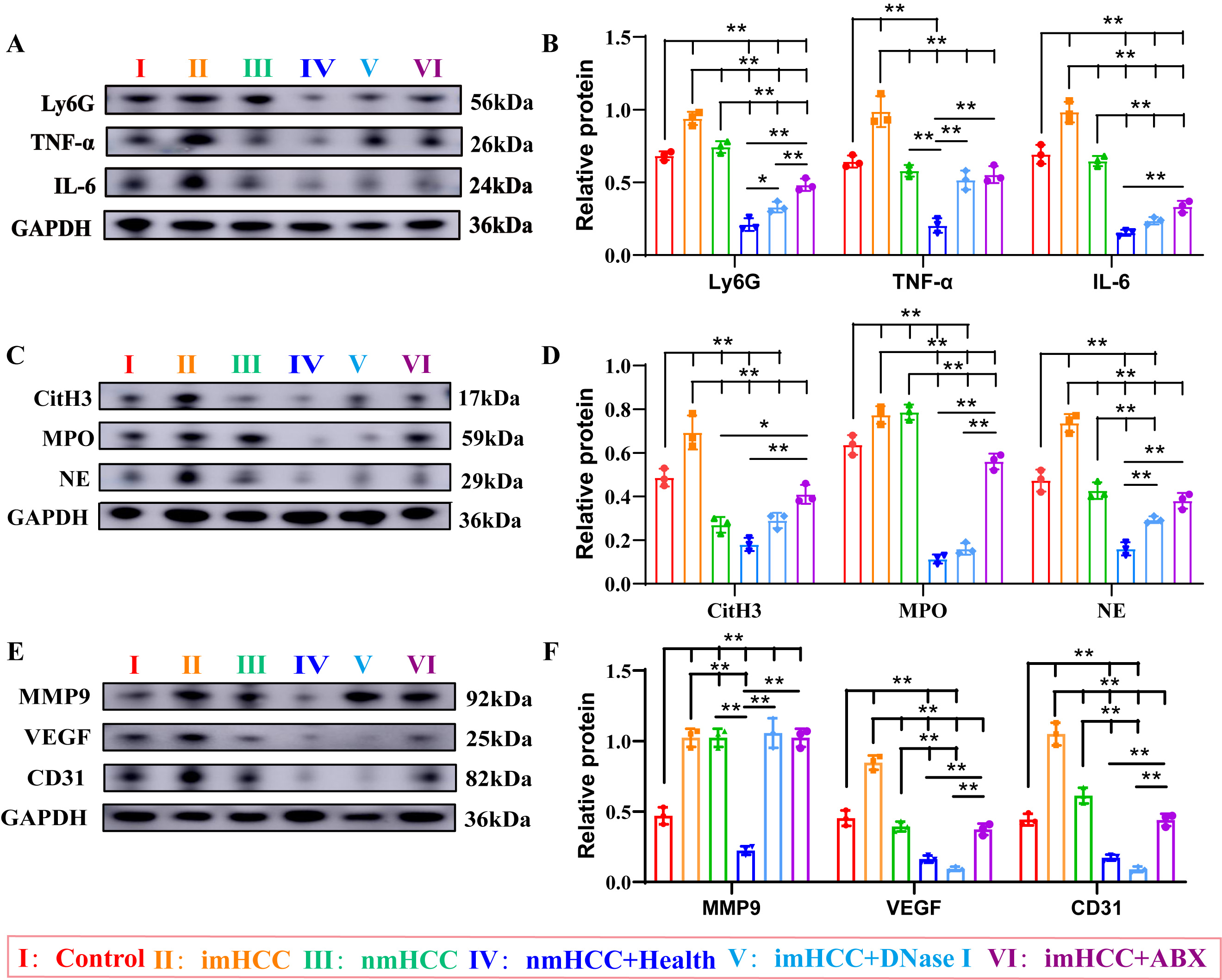


Figure S6. Western blot was performed to detect the expression of neutrophil inflammatory activation markers, NETs formation markers, and vascular growth factors in each group of mice. A-B. Protein expression of neutrophilic inflammatory markers Ly6G, inflammatory cytokines TNF-α, and IL-6 in each group of mice. C-D. Protein expression of NETs formation markers (CitH3, MPO, and NE) in each group of mice. E-F. Protein expression of vascular growth factors VEGF, MMP9 and CD31 in each group of mice. NETs: neutrophil extracellular traps, Ly6G: lymphocyte antigen 6 complex, locus G; TNF-α: Tumour necrosis factor-α; IL-6: interleukin 6; CitH3: Citrullinated histone H3; MPO: Myeloperoxidase; VEGF: vascular endothelial growth factor; MMP9: Matrix metalloproteinase-9; IHC staining: Immunohistochemical staining; IF staining: immunofluorescence staining. **P* <0.05; ***P* <0.01.

**Figure** **S7. DNase I elimination of NETs formation inhibits HCC intrahepatic metastasis**


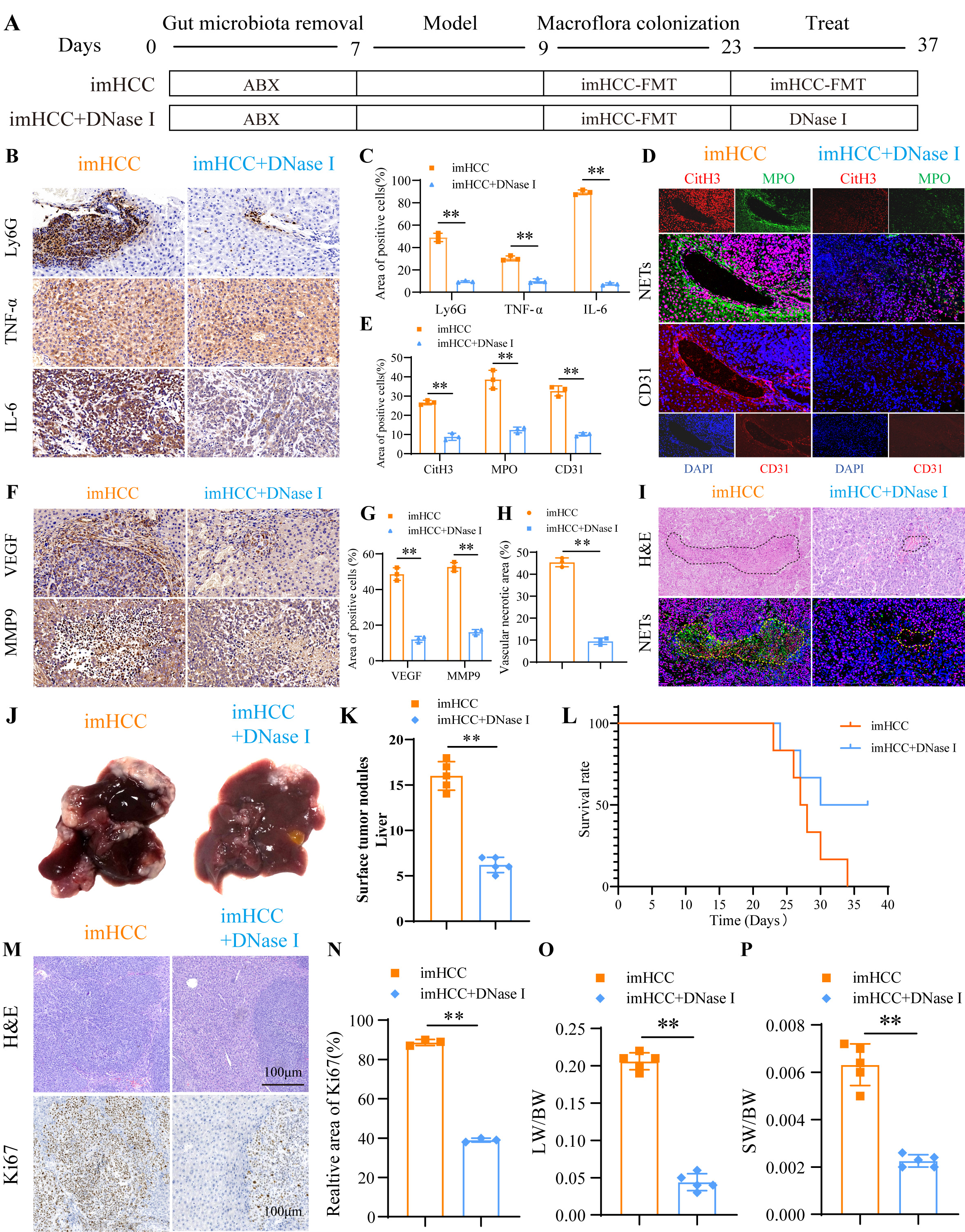


Figure S7. DNase I elimination of NETs formation inhibits HCC intrahepatic metastasis. A. Experimental Design Workflow. B-C. Protein expression of neutrophilic inflammatory markers Ly6G, inflammatory cytokines TNF-α, and IL-6 in each group of mice. D-E. Co-expression of NETs formation markers (CitH3 and MPO) with CD31 in each group of mice. F-G. Protein expression of VEGF and MMP9 in each group of mice. H and I. Co-expression of necrotic foci and NETs Representative images of intrahepatic metastatic tumors each group mice. K. Quantification of intrahepatic metastatic tumors in each group mice. J. Representative images of intrahepatic metastatic tumors in each group of mice. K. Quantification of intrahepatic metastatic tumors in each group of mice. L. The survival curves in each group of mice. M. Representative images of H&E staining and Ki67 IHC staining in each group of mice. O. The ratio of LW/BW in each group of mice. P. The ratio of SW/BW in each group of mice. The aforementioned experiments were conducted using an animal model established with Huh-7 Luc1 cells. FMT: faecal microbiota transplantation; NETs: neutrophil extracellular traps, Ly6G: lymphocyte antigen 6 complex, locus G; TNF-α: Tumour necrosis factor-α; IL-6: interleukin 6; CitH3: Citrullinated histone H3; MPO: Myeloperoxidase; VEGF: vascular endothelial growth factor; MMP9: Matrix metalloproteinase-9; IHC staining: Immunohistochemical staining; IF staining: immunofluorescence staining; LW: Liver weight; SW: Spleen weight; BW: Body weight. **P* <0.05; ***P* <0.01.

**Figure S8. Healthy FMT and DNase I can inhibit HCC intrahepatic metastasis.**


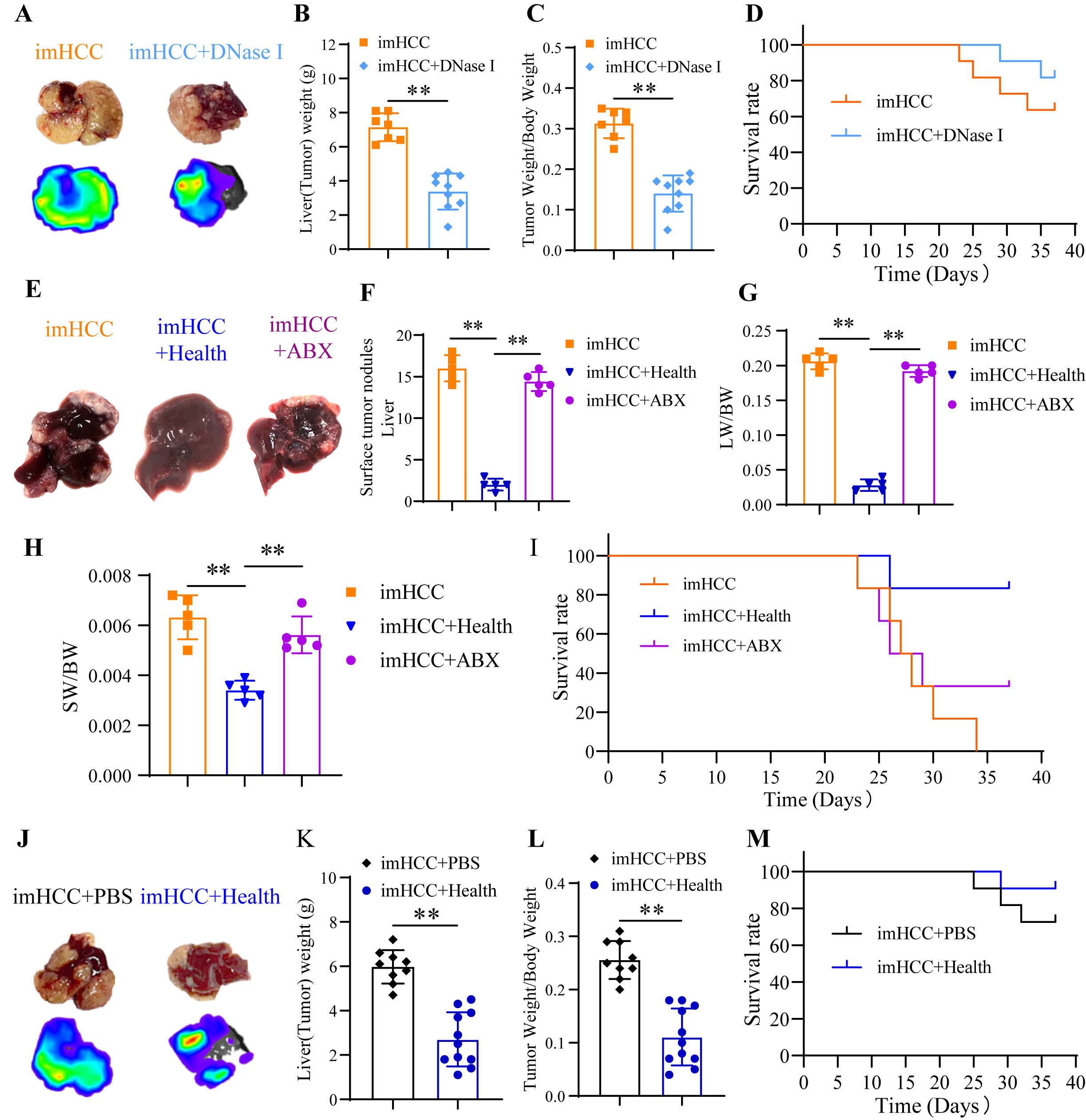


Figure S8. Healthy FMT and DNase I can inhibit HCC intrahepatic metastasis. A. Representative images of intrahepatic metastatic tumours and bioluminescence imaging between imHCC and imHCC+DNase I group. B. Liver(Tumor) weight of mice between imHCC and imHCC+DNase I group. C. Ratio of Liver(Tumor) weight/Body weight of mice between imHCC and imHCC+DNase I group. D. Survival curves between imHCC and imHCC+DNase I group of mice. E. Representative images of intrahepatic metastatic tumours for imHCC, imHCC+Health and imHCC+ABX group. F. Quantification of intrahepatic metastatic tumours for imHCC, imHCC+Health and imHCC+ABX group. G. Ratio of LW/BW for imHCC, imHCC+Health and imHCC+ABX group of mice. H. Ratio of SW/BW for imHCC, imHCC+Health and imHCC+ABX group of mice. I. Survival curves for imHCC, imHCC+Health and imHCC+ABX group of mice. J. Representative images of intrahepatic metastatic tumours and bioluminescence imaging between imHCC+PBS and imHCC+Health group. K. Liver(Tumor) weight of mice between imHCC+PBS and imHCC+Health group. L. Ratio of Liver(Tumor) weight/Body weight of mice between imHCC+PBS and imHCC+Health group. M. Survival curves between imHCC+PBS and imHCC+Health group of mice. The animal models in Figures A-D and J-M were constructed using MHCC97H Luc1 cells, and the animal models in Figures D-C were constructed using Huh-7 Luc1 cells. imHCC: Intrahepatic metastatic HCC; nmHCC: Non-metastatic HCC; LW: Liver weight; SW: Spleen weight; BW: Body weight. **P* <0.05; ***P* <0.01.

**Figure S9.** **Healthy FMT improved the dysregulated gut microbiota in HFA-intrahepatic metastasis HCC mice.**


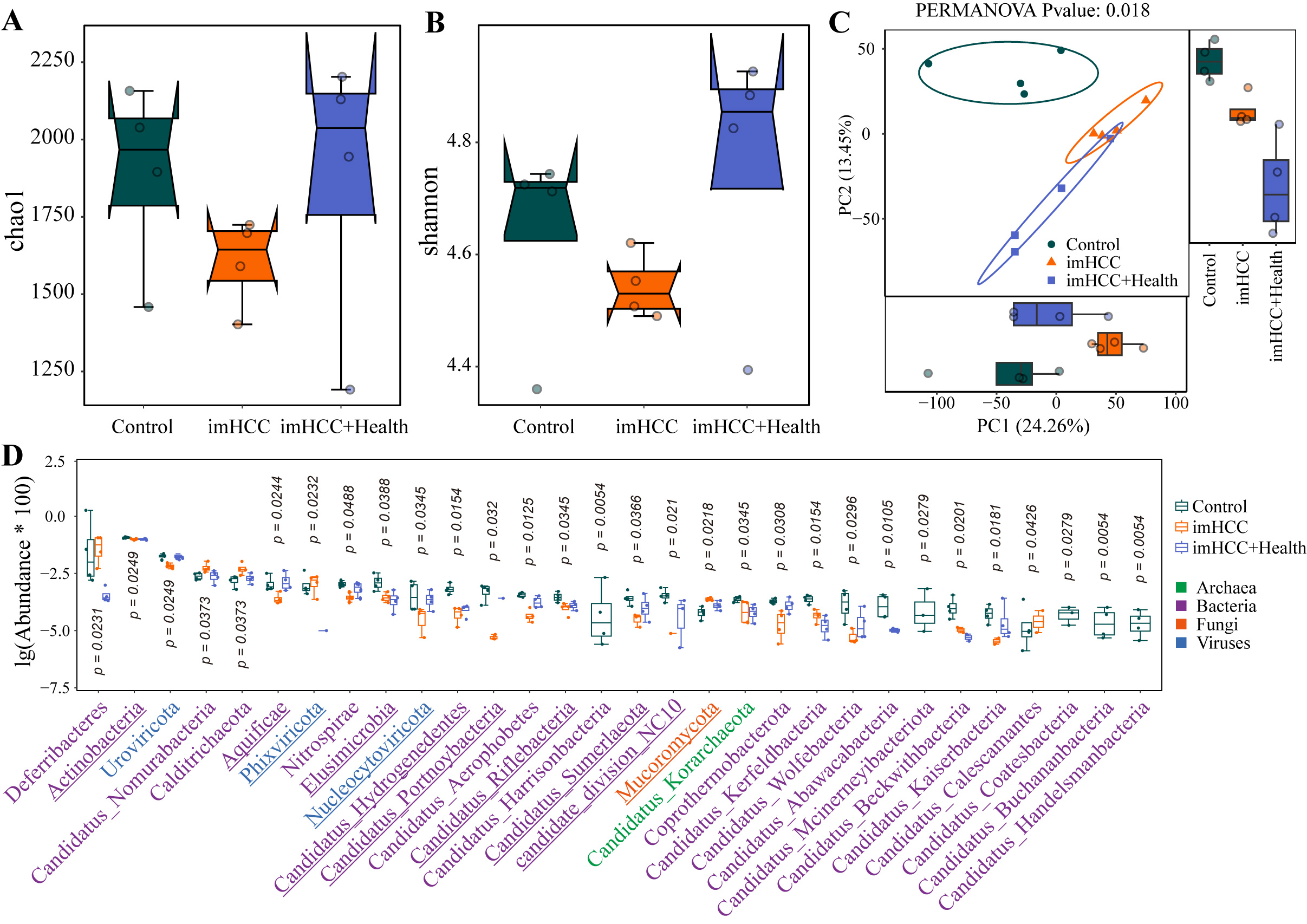


Figure S9. Healthy fecal microbiota transplantation improved the dysregulated gut microbiota in HFA-intrahepatic metastasis HCC mice. (A and B) Chao1 index (A) and Shannon index (B) analyses of gut microbiota in Control, imHCC, and imHCC+Health groups of mice; (C) PCA of gut microbiota in each group of mice; (D and E) Relative abundance comparison of the top 30 different microbiota at the phylum levels in each group of mice. FMT: faecal microbiota transplantation; HFA: human flora-associated; imHCC: intrahepatic metastatic HCC；nmHCC: non-metastatic HCC.
